# Renal autocrine neuropeptide FF (NPFF) signaling regulates blood pressure

**DOI:** 10.1038/s41598-024-64484-9

**Published:** 2024-07-04

**Authors:** Hewang Lee, Bibhas Amatya, Van Anthony M. Villar, Laureano D. Asico, Jin Kwon Jeong, Jun Feranil, Shaun C. Moore, Xiaoxu Zheng, Michael Bishop, Jerald P. Gomes, Jacob Polzin, Noah Smeriglio, Pedro A. S. Vaz de Castro, Ines Armando, Robin A. Felder, Ling Hao, Pedro A. Jose

**Affiliations:** 1 Division of Renal Diseases and Hypertension, Department of Medicine, The George Washington University School of Medicine and Health Sciences, 2300 Eye Street, NW, Washington, DC 20052 USA; 2 Department of Medicine, University of Maryland School of Medicine, Baltimore, MD 21201 USA; 3 Department of Pharmacology and Physiology, The George Washington University School of Medicine and Health Sciences, Washington, DC 20052 USA; 4 Department of Chemistry, Columbian College of Arts and Sciences, The George Washington University, Washington, DC 20052 USA; 5 Department of Pathology, University of Virginia Health Sciences Center, Charlottesville, VA 22908.5 USA

**Keywords:** Blood pressure, Brain, Dopamine D1-like receptor, Kidney, Neuropeptide FF, Biochemistry, Cell biology, Genetics, Physiology, Endocrinology, Medical research, Molecular medicine, Nephrology

## Abstract

The kidney and brain play critical roles in the regulation of blood pressure. Neuropeptide FF (NPFF), originally isolated from the bovine brain, has been suggested to contribute to the pathogenesis of hypertension. However, the roles of NPFF and its receptors, NPFF-R1 and NPFF-R2, in the regulation of blood pressure, via the kidney, are not known. In this study, we found that the transcripts and proteins of NPFF and its receptors, NPFF-R1 and NPFF-R2, were expressed in mouse and human renal proximal tubules (RPTs). In mouse RPT cells (RPTCs), NPFF, but not RF-amide-related peptide-2 (RFRP-2), decreased the forskolin-stimulated cAMP production in a concentration- and time-dependent manner. Furthermore, dopamine D1-like receptors colocalized and co-immunoprecipitated with NPFF-R1 and NPFF-R2 in human RPTCs. The increase in cAMP production in human RPTCs caused by fenoldopam, a D1-like receptor agonist, was attenuated by NPFF, indicating an antagonistic interaction between NPFF and D1-like receptors. The renal subcapsular infusion of NPFF in C57BL/6 mice decreased renal sodium excretion and increased blood pressure. The NPFF-mediated increase in blood pressure was prevented by RF-9, an antagonist of NPFF receptors. Taken together, our findings suggest that autocrine NPFF and its receptors in the kidney regulate blood pressure, but the mechanisms remain to be determined.

## Introduction

Hypertension is caused by the complex interplay among environment, lifestyle, and genetics^1,2^, in which gene–gene interactions play critical roles in its development^1,2^. However, genome-wide association studies in hypertension have only revealed a small fraction of genetically regulated blood pressure variability^3^. The specific genes involved in kidney-mediated hypertension remain to be fully understood.

Dopamine, a neurotransmitter first identified in the brain, is an important regulator of systemic blood pressure by its actions on fluid and electrolyte balance, mediated by the kidney^4–7^. The kidney synthesizes dopamine from circulating and filtered L-3,4-dihydroxyphenyl-alanine, independently of its innervation^4^. Dopamine accounts for ≥ 50% of renal sodium excretion under conditions of moderate sodium excess^4–6^. Renal dopamine exerts its actions via two subfamilies of G protein-coupled receptors, D1-like (D_1_R and D_5_R) and D2-like (D_2_R, D_3_R, and D_4_R) receptors^4–6^. The D1-like receptors couple to the stimulatory G protein, Gαs, and activate adenylate cyclase whereas the D2-like receptors couple to the inhibitory G protein, Gαi, and inhibit adenylate cyclase^4–6^. The impairment of D1-like receptor function underlies, in part, the increased blood pressure observed in several mouse models of hypertension and some humans with hypertension^4–8^. Studies have shown that the germline deletion of either *Drd1*^7^ or *Drd5*^8^ gene in mice also results in hypertension. Furthermore, D1-like receptors regulate blood pressure, in part, by counteracting the effects of pro-hypertensive factors, such as angiotensin II and catecholamines^4–8^.

Neuropeptide FF (NPFF, FLFQPQRF-NH2), a mammalian-amidated neuropeptide originally isolated from bovine brain, is a pain-modulating peptide, with anti-opioid activity in the rat^9^. NPFF, which shares the same *NPFF* gene with neuropeptide AF, participates in cardiovascular regulation, energy metabolism, food consumption, immunity, nerve injury repair, and pain modulation^10^. NPFF exerts its effects through its two receptors, NPFF-R1 (GPR147) and NPFF-R2 (GPR74)^11,12^. The ability of guanine nucleotides to inhibit NPFF binding to its receptors suggests that both NPFF-R1 and NPFF-R2 receptors are coupled to G proteins^10^. Both NPFF-R1 and NPFF-R2 preferentially couple to Gαi/o protein and inhibit adenylate cyclase activity^13^. However, NPFF-R2 can also couple to Gαs and stimulate adenylate cyclase activity in mouse cerebellum, olfactory bulb, and spinal cord^14^. In addition to the central nervous system (CNS), NPFF and its receptors, NPFF-R1 and NPFF-R2, are also present in peripheral tissues, including the kidney^10–12^.

NPFF may participate in the regulation of blood pressure because it is present in the cardiovascular regulatory center in the hypothalamus^15^. The intracerebroventricular^16^, intranuclear tractus solitarius^17^, or intrathecal^18^ administration of NPFF increases blood pressure, indicating that NPFF in the central nervous system increases blood pressure. The intravenous administration of NPFF also increases blood pressure^19^ although it cannot cross the blood-brain barrier^20^. Therefore, both central and peripheral mechanisms contribute to the NPFF-mediated increase in blood pressure^15–22^.

The NPFF network in the hypothalamus is impaired in hypertensive patients^23^ suggesting that the interaction of the NPFF system with other neurotransmitter system (s) could play an important role in the regulation of blood pressure. The dopaminergic system in the kidney is known to regulate blood pressure^4–8,24,25^, however, it is not known whether NPFF can regulate blood pressure in the kidney, and whether or not there is a functional interaction between NPFF and the dopaminergic system in the kidney.

The main objective of this study is to determine the presence of the NPFF system in the kidney, the physical and functional interaction between the NPFF system in renal proximal tubule cells (RPTCs) by determining their cAMP production, a known signaling pathway mediated by dopamine receptors, and the renal-mediated blood pressure regulation by NPFF and its receptors, NPFF-R1 and NPFF-R2.

## Results

### Autocrine NPFF and its receptors in the kidney

**Figure 1:**
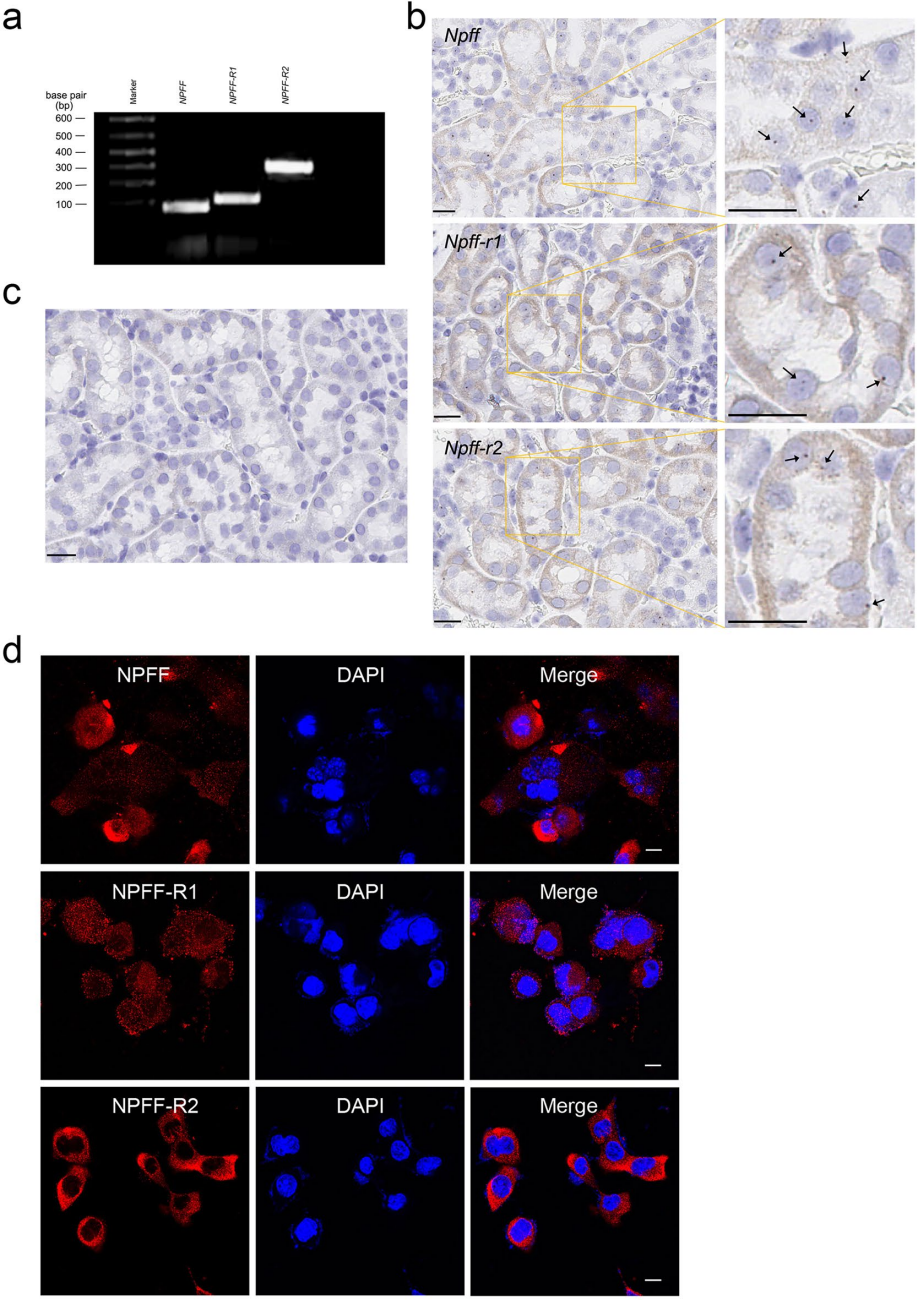
Gene expression of *NPFF*(*Npff*) and its receptors in human and mouse kidneys. (**a**) Agarose gel electrophoresis of RT-PCR products of *NPFF*, *NPFF-R1,* and *NPFF-R2* in human renal proximal tubule cells (hRPTCs). (**b**) Gene expressions of *Npff*, *Npff-r1*, and *Npff-r2* in the RPTs of C57BL/6 mice analyzed by RNAScope. The mouse kidney sections were hybridized using specific *Npff*, *Npff-r1*, and *Npff-r2* probes (Advanced Cell Diagnostics); *Npff*, *Npff-r1*, and *Npff-r2* RNA appear as deep brown dots, some of them indicated by arrows (right panel). (**c**) Negative control images: no visible signals can be seen in kidney sections hybridized using a non-specific probe, *dapB*, a bacterial gene (Advanced Cell Diagnostics). Bar scale, 20 μm. (**d**) mRPTCs were prepared and stained as described in the Materials and Methods section. The mRPTCs were incubated with anti-FMRF (for NPFF), anti-NPFF-R1, and anti-NPFF-R2 antibodies, as indicated (left panel), and counterstained for DNA with DAPI (4′,6-diamidino-2-phenylindole, middle panel). The merged images (right panel) show possible nuclear staining of NPFF and its receptors. Bar scale, 10 μm.

Previous studies have shown that *Npff* and its receptors are expressed in the central nervous system and peripheral tissues^10–12,26–28^. We found that in the brain (Supplementary Fig. S1), NPFF-R1 was expressed in multiple hypothalamic nuclei, including the organum vasculosum of the lamina terminalis (OVLT), supraoptic nucleus (SON), paraventricular nucleus (PVN), arcuate nucleus (ARC), and ventromedial hypothalamus (VMH), and in non-hypothalamic regions, such as the hippocampus (HP) and cerebral cortex (CTX), but absent in the dorsomedial hypothalamus (DMH) and piriform cortex (Pir). By contrast, NPFF-R2 was widely and strongly expressed in hypothalamic nuclei, including the OVLT, SON, PVN, ARC, and VMH, and in non-hypothalamic regions such as the HP, Pir, and CTX. In the kidney, we specifically examined their expression in human RPTCs (hRPTCs) and mouse RPTCs (mRPTCs). As determined by RT-PCR (Fig. 1a), the transcripts of *NPFF* and its receptors *NPFF-R1* and *NPFF-R2* were expressed in hRPTCs. The mouse mRNAs of *Npff* and its receptors *Npff-r1* and *Npff-r2*, determined by RNA in situ hybridization (RNAscope), were positively stained when hybridized with their specific probes in the proximal tubule of C57BL/6 mouse kidney (Fig. 1b), while a negative control probe displayed no staining (Fig. 1c). NPFF, NPFF-R1, and NPFF-R2 proteins were also expressed in mRPTCs (Fig. 1d) and hRPTCs (Supplementary Fig. S2); Overall, the staining of NPFF-R1 was weaker than that of NPFF-R2.

**Figure 2:**
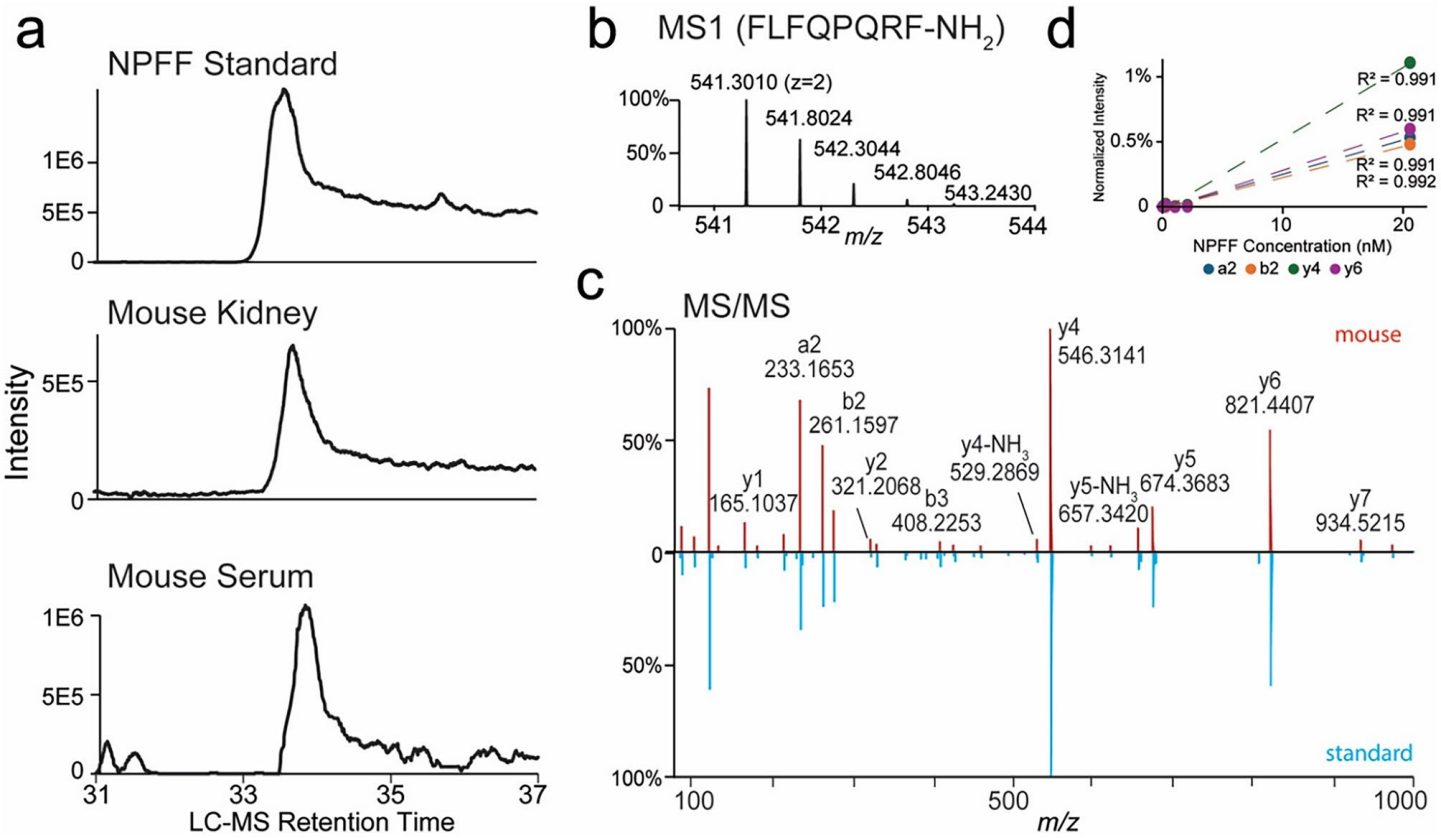
Analysis of NPFF peptide by targeted LC–MS/MS. (**a**) Extracted LC–MS ion chromatograms (m/z 541.3010) of NPFF peptide standard and NPFF extracted from the mouse kidney and serum. (**b**) MS spectrum of NPFF peptide. (**c**) MS/MS peptide fragmentation spectrum of NPFF peptide detected in mouse serum (red) versus standard (blue). (**d**) NPFF calibration curves using a2, b2, y4, and y6 fragment ions based on the targeted parallel reaction monitoring (PRM) assay.

**Figure 3:**
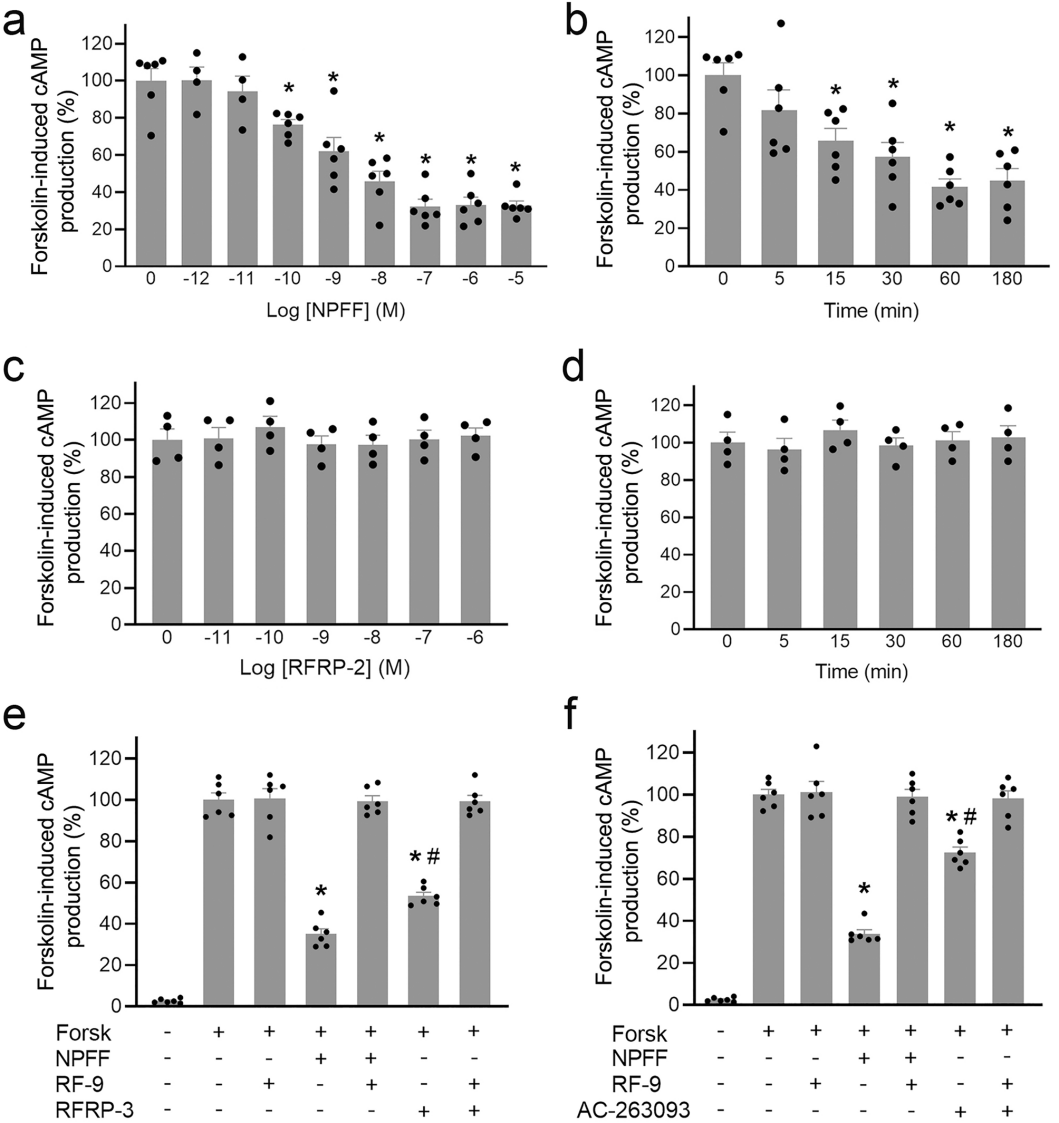
Inhibition of cAMP production by NPFF in mRPTCs. (**a**) mRPTCs were exposed to the indicated concentrations of NPFF for 15 min, followed by 10 µM forskolin for 30 min. (**b**) mRPTCs were exposed to 10^−7^ M NPFF at the indicated time points, followed by 10 µM forskolin for 30 min. n = 4. * *p* < 0.05 versus 0 M or 0 min, one-way ANOVA, Newman-Keuls test. (**c**) mRPTCs were treated with RFRP-2, an RF-amide with no known function, at the indicated concentrations for 15 min, n = 4. (**d**) mRPTCs were treated with RFRP-2 (10^−7^ M) at the indicated time points, followed by 10 µM forskolin for 30 min (right). n = 4. * *p* < 0.05 versus 0 M or 0 min, one-way ANOVA, Newman-Keuls test. (**e**) mRPTCs were exposed to RFRP-3, an NPFF-R1 agonist, in the absence or presence of RF-9, a dual NPFF-R1 and NPFF-R2 antagonist, as indicated, for 15 min, followed by 10 µM forskolin (Forsk) for 30 min. (**f**) mRPTCs were exposed to AC-263093, an NPFF-R2 agonist, in the absence or presence of RF9, as indicated, for 15 min, followed by 10 µM forskolin (Forsk) for 30 min. n = 6, * *p* < 0.05 versus Forsk alone, # *p* < 0.05 versus Forsk plus NPFF, one-way ANOVA, Newman-Keuls test. NPFF, an agonist for both NPFF-R1 and NPFF-R2, served as positive control to inhibit Forsk-induced increase in cAMP production in (**a**) and (**b**).

The presence of NPFF in the mouse serum and kidney was confirmed by liquid chromatography-tandem mass spectrometry (LC–MS/MS) (Fig. 2); the NPFF concentrations were 0.936 ± 0.22 pmol/g of tissue (n = 3) in the kidney and 541 ± 32 pmol/L (n = 3) in the serum of C57BL/6 mice. Next, we determined whether NPFF produced in mRPTCs is functional. As shown in Fig. 3, NPFF inhibited the forskolin-induced increase in cAMP production in mRPTCs in a concentration- (Fig. 3a) and time- (Fig. 3b) dependent manner. By contrast, RFRP-2, an RF-amide-related peptide with low affinity to NPFF receptors^28^, had no effect on forskolin-induced cAMP production (Fig. 3c,d). The ability of NPFF, but not RFRP-2, to decrease the forskolin-induced increase in cAMP level measured in cell culture supernatants (Fig. 3) was also observed in mRPTC lysates (Supplementary Fig. S3).

NPFF binds to both NPFF-R1 and NPFF-R2^11^. To determine further the role of NPFF in the inhibition of cAMP production, we used RF-9, a dual NPFF-R1 and NPFF-R2 antagonist^29^, RFRP-3, an NPFF-R1 agonist^30^, and AC-263093, an NPFF-R2 agonist^31^. RFRP-3 (10^−7^ M) inhibited the forskolin-stimulated cAMP production (Fig. 3e), which was reversed by pretreatment with RF-9 (10^−5^ M). Similarly, AC-263093 (10^−6^ M) inhibited the forskolin-stimulated cAMP production (Fig. 3f), which was also reversed by pretreatment with RF-9 (10^−5^ M). Of note, RF-9, by itself, had no effect. This highlights that, unlike in GnRH neurons^32^, in mRPTCs, RF-9 has no effect on the kisspeptin receptor, indicating receptor specificity at least in RPTCs.

### Interaction between NPFF and dopaminergic system in the kidney

**Figure 4:**
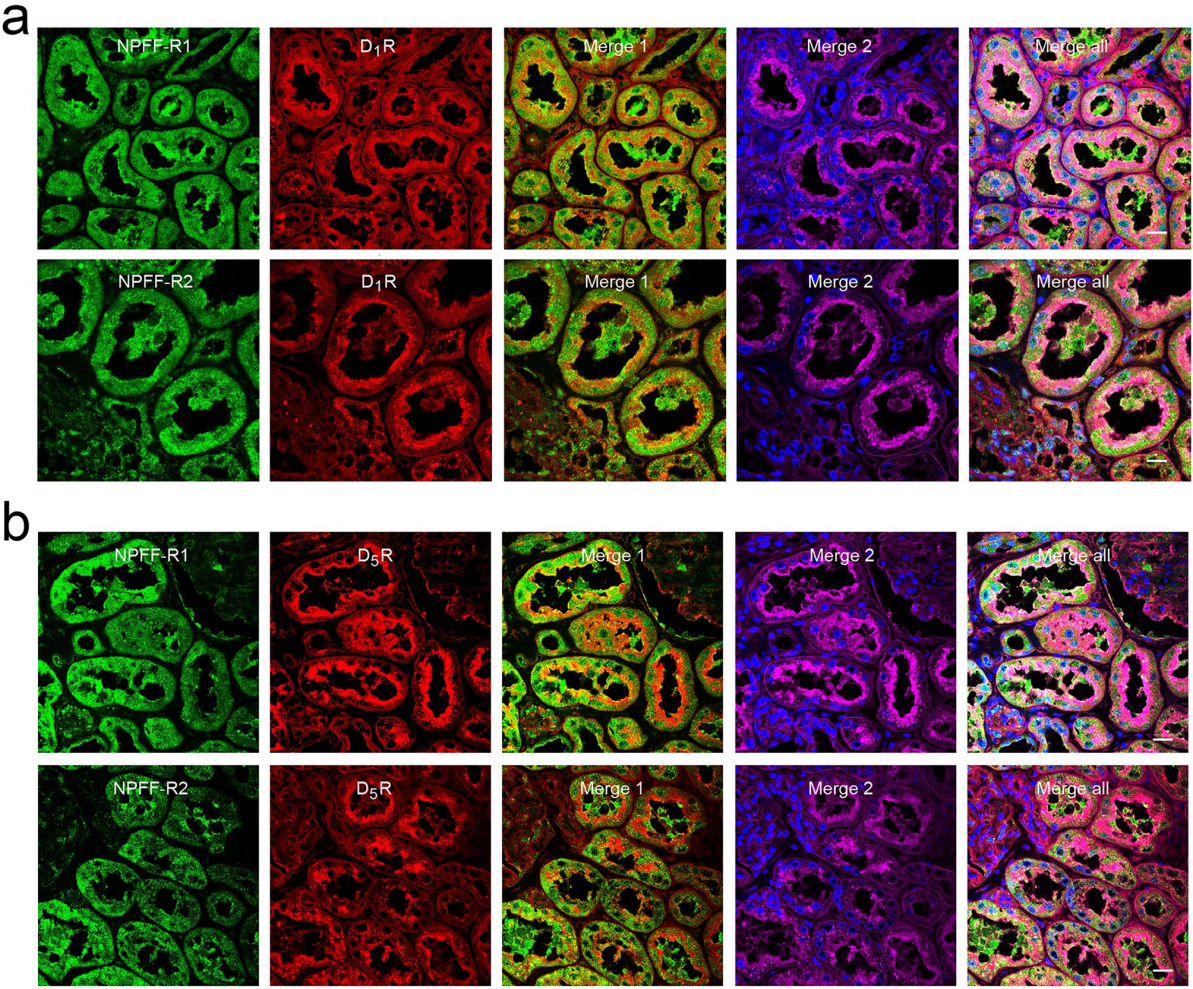
Colocalization of NPFF receptors with D1-like receptors (D_1_R and D_5_R) in human kidney sections. (**a**) Strong co-localization of NPFF-R1 and NPFF-R2 with D_1_R in human kidney sections. (**b**) Strong co-localization of NPFF-R1 with D_5_R but minimal colocalization of NPFF-R2 with D_5_R in human kidney sections. NPFF-R1 or NPFF-R2, green; D_1_R or D_5_R, red; wheat germ agglutinin (WGA, plasma membrane marker), magenta; DAPI (4′,6-diamidino-2-phenylindole), blue. Merge 1, NPFF-R1 (or NPFF-R2) with D_1_R (or D_5_R), the colocalization of NPFF receptors with D_1_R or D_5_R is denoted in yellow; Merge 2, WGA with DAPI. Bar scale, 20 µm.

**Figure 5:**
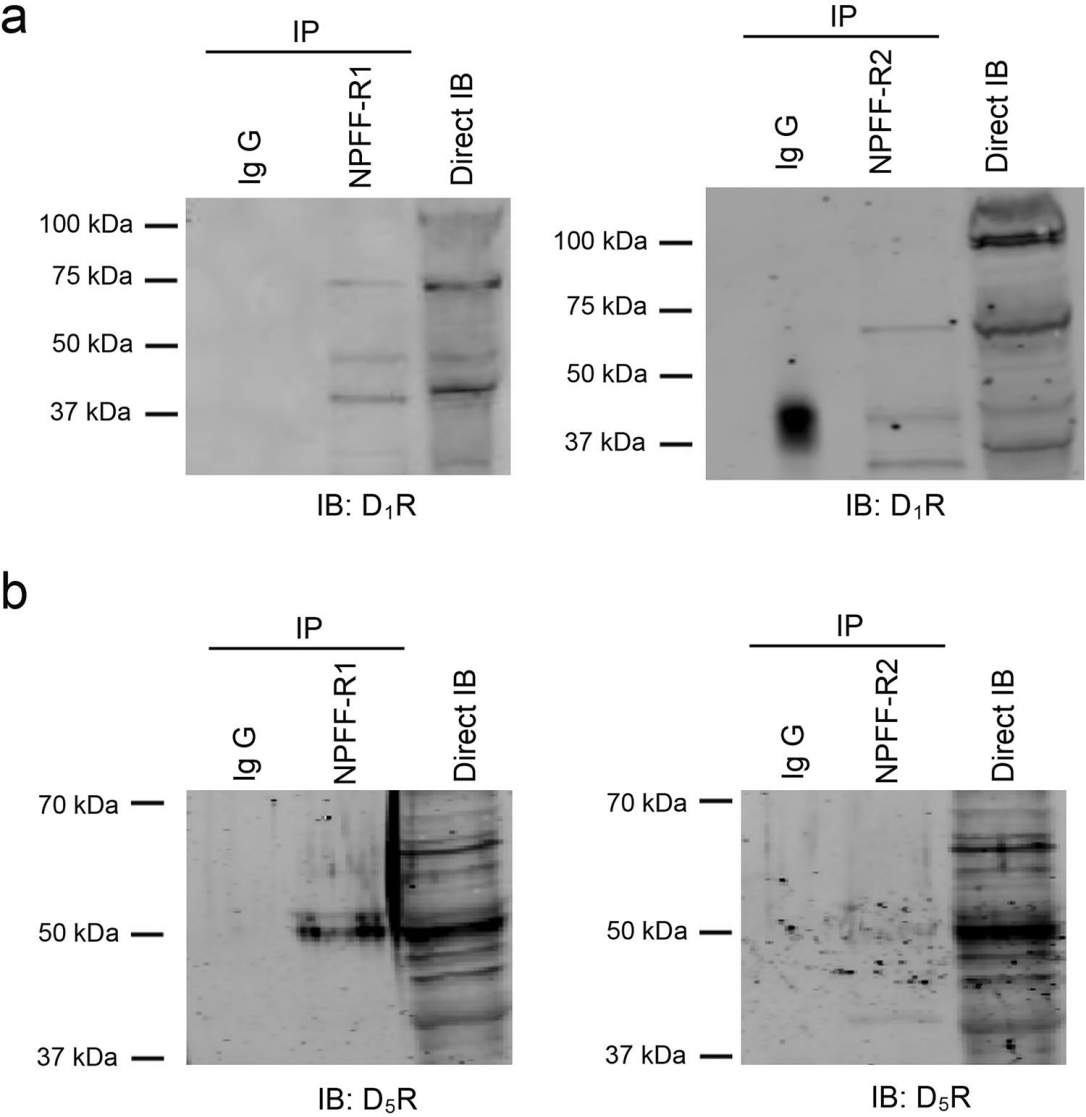
Co-immunoprecipitation of NPFF receptors with D1-like receptors (D_1_R and D_5_R) in hRPTCs. (**a**) Co-immunoprecipitation of NPFF-R1 and NPFF-R2 with D_1_R in hRPTCs. (**b**) Co-immunoprecipitation of NPFF-R1 but not NPFF-R2 with D_5_R in hRPTCs. hRPTC lysates were immunoprecipitated (IP) with anti-NPFF-R1 or anti-NPFF-R2 antibodies coupled to Dynabeads for 4 h at 4 °C. The protein complexes bound to the beads were eluted and separated by SDS-PAGE, transferred onto nitrocellulose membranes, and immunoblotted (IB) with anti-D_1_R (**a**) or anti-D_5_R (**b**) antibodies, as indicated. The expected bands for D_1_R and D_5_R are at 70 kDa and 55 kDa, respectively. Normal IgG was used for negative control and immunoblotting of D_1_R or D_5_R in cell lysates for positive control.

Dopamine, initially found in the brain, stimulates adenylyl cyclase through D1-like receptors in hRPTCs or mRPTCs^4–6^. Therefore, we investigated whether there is an interaction between the NPFF and D1-like dopaminergic systems in the kidney. In hRPTCs, both NPFF-R1 and NPFF-R2 co-localized with D_1_R (Supplementary Fig. S4a). NPFF-R1 also co-localized with D_5_R, but NPFF-R2 had minimal co-localization with D_5_R (Supplementary Fig. S4b). Consistent with the hRPTC studies, in human kidney sections both NPFF-R1 and NPFF-R2 also co-localized with D_1_R (Fig. 4a); NPFF-R1 also co-localized with D_5_R while NPFF-R2 minimally co-localized with D_5_R (Fig. 4b). Furthermore, we found that both D_1_R (Fig. 5a, left panel) and D_5_R (Fig. 5b, left panel) co-immunoprecipitated with anti-NPFF-R1 antibody in hRPTC lysates. However, NPFF-R2 co-immunoprecipitated with the D_1_R (Fig. 5a, right panel) but not the D_5_R (Fig. 5b, right panel) in hRPTC lysates. These results suggested the potential interaction between NPFF receptors and D1-like receptors, specifically NPFF-R1 with both D_1_R and D_5_R but NPFF-R2 only with D_1_R, in the hRPTCs.

### Antagonism between NPFF and dopaminergic systems in the kidney

**Figure 6:**
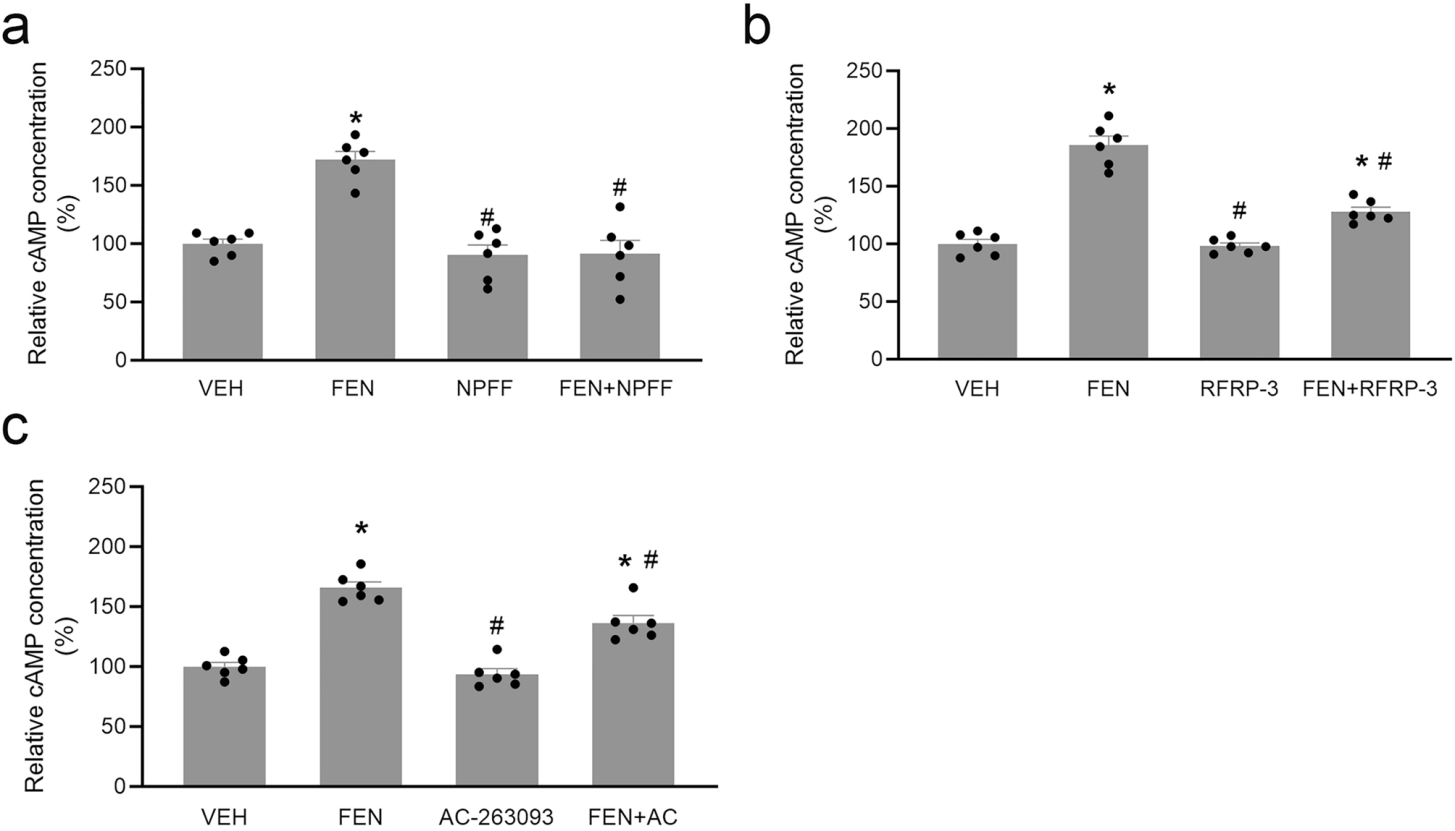
Antagonism between NPFF receptors and D1-like receptors on cAMP production. hRPTCs were treated with fenoldopam (FEN, 10^−7^ M), a D1-like receptor agonist, without or with NPFF (10^−11^ M) (**a**), RFRP-3 (10^−10^ M) (**b**), or AC-263093 (10^−7^ M) (**c**) for 30 min. VEH, vehicle. n = 6/group, * *p* < 0.05 versus VEH, # *p* < 0.05 versus FEN, One-way ANOVA, Newman-Keuls test.

We next investigated the potential physiological/pathophysiological effect of the interaction between the NPFF and the D1-like dopaminergic systems in the kidney. As expected, the D1-like receptor agonist, FEN^4–6^, increased intracellular cAMP production in hRPTCs; NPFF (10^−11^ M) impaired the stimulatory effect of FEN at a concentration which, by itself, did not affect cAMP concentration (Fig. 6a). Similarly, the NPFF-R1 agonist, RFRP-3 (Fig. 6b) and NPFF-R2 agonist, AC-263093 (Fig. 6c), by themselves, did not induce changes in the cAMP concentration but they attenuated the stimulatory effect of FEN on intracellular cAMP concentration, indicating that both NPFF-R1 and NPFF-R2 can antagonize the stimulatory effect of FEN on cAMP production.

### NPFF and the protein expression of renal sodium transporters

**Figure 7:**
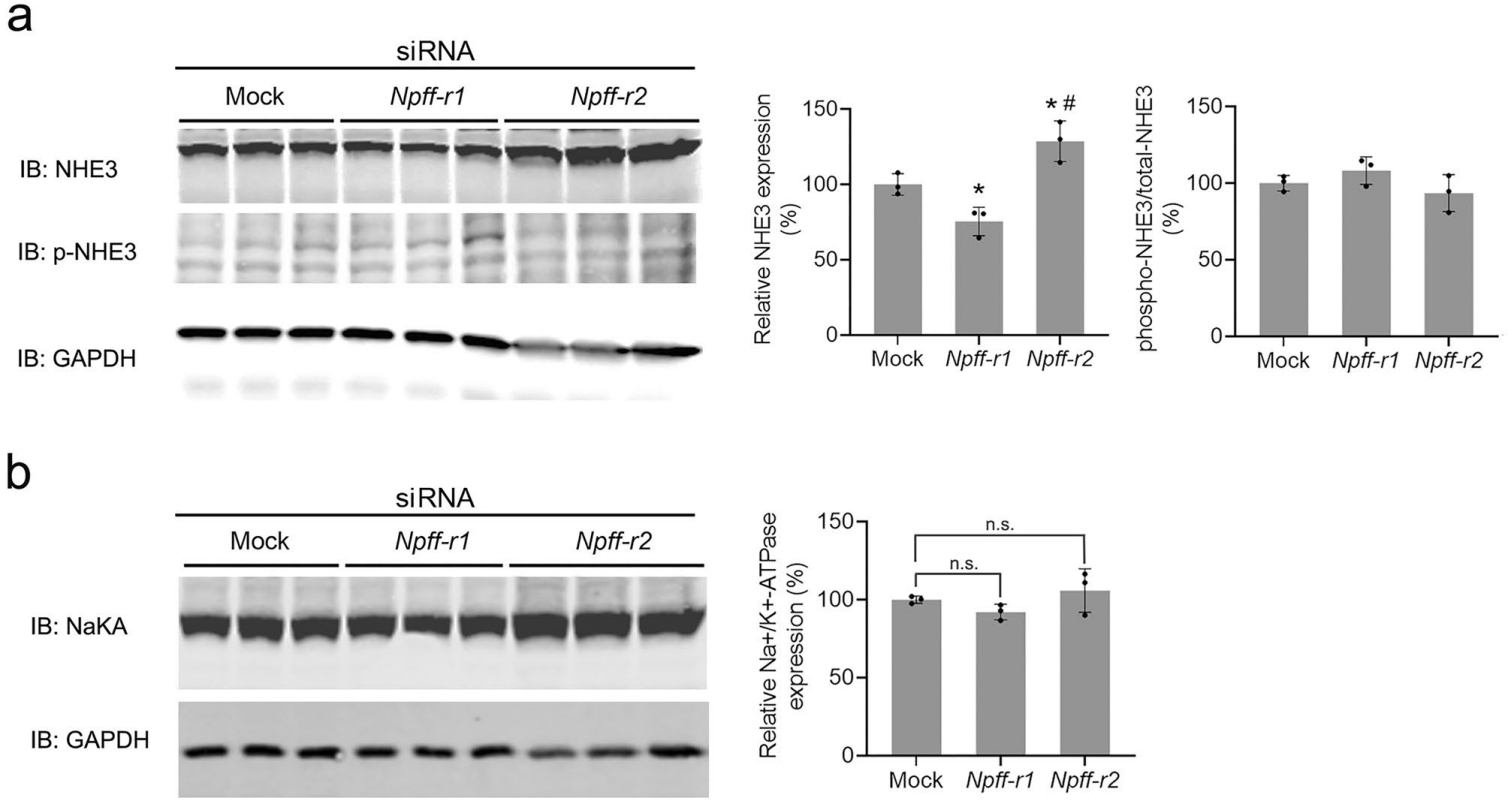
Renal protein expression of Na^+^/H^+^ exchanger type 3 (**a**) and Na^+^/K^+^-ATPase (**b**) after the renal subcapsular infusion of mock, *Npff-r1* siRNA, or *Npff-r2* siRNA in C57BL/6 mice fed a normal salt diet. Left panels, representative immunoblots of the protein expressions of NHE3 (upper panel in **a**), phospho-NHE3 (middle panel in **a**)*,* and Na^+^/K^+^-ATPase (upper panel in **b**) as indicated. Immunoblots of GAPDH served as loading control. Densitometric analysis of immunoblots. n = 3/group, * *p* < 0.05 versus Mock, # *p* < 0.05 versus *Npff-r1* siRNA, one-way ANOVA, Newman-Keuls test.

As determined by immunoblotting, the renal protein expression of the Na^+^/H^+^ exchanger type 3 (NHE3), was slightly decreased by the renal subcapsular infusion of *Npff-r1* siRNA (Fig. 7a). By contrast, the NHE3 protein expression was markedly increased by the renal subcapsular infusion of *Npff-r2* siRNA in C57BL/6 mice fed a normal salt diet (Mock: 100.0 ± 7.2%, *Npff-r1* siRNA: 75.4 ± 9.4%, *Npff-r2* siRNA: 128.7 ± 13.5%, n = 3; *P* < 0.05) (Fig. 7a). There was no effect on NHE3 phosphorylation (Fig. 7a). The protein expression of Na^+^/K^+^-ATPase tended to be decreased by the renal subcapsular infusion of *Npff-r1* siRNA and tended to be increased by the renal subcapsular infusion of *Npff-r2* siRNA in C57BL/6 mice fed a normal salt diet (Mock: 100 ± 2.3%, *Npff-r1* siRNA: 92.1 ± 5.0%, *Npff-r2* siRNA: 105.8 ± 13.9%, n = 3; *P* = 0.229) (Fig. 7b). These results indicate that in mouse kidneys, NPFF-R1 stimulates while NPFF-R2 inhibits NHE3 expression. These findings suggest that NPFF may not affect overall renal sodium handling via NHE3 and Na^+^/K^+^-ATPase, in mice that are fed a normal sodium diet.

### Effect of NPFF on sodium excretion and blood pressure

**Figure 8:**
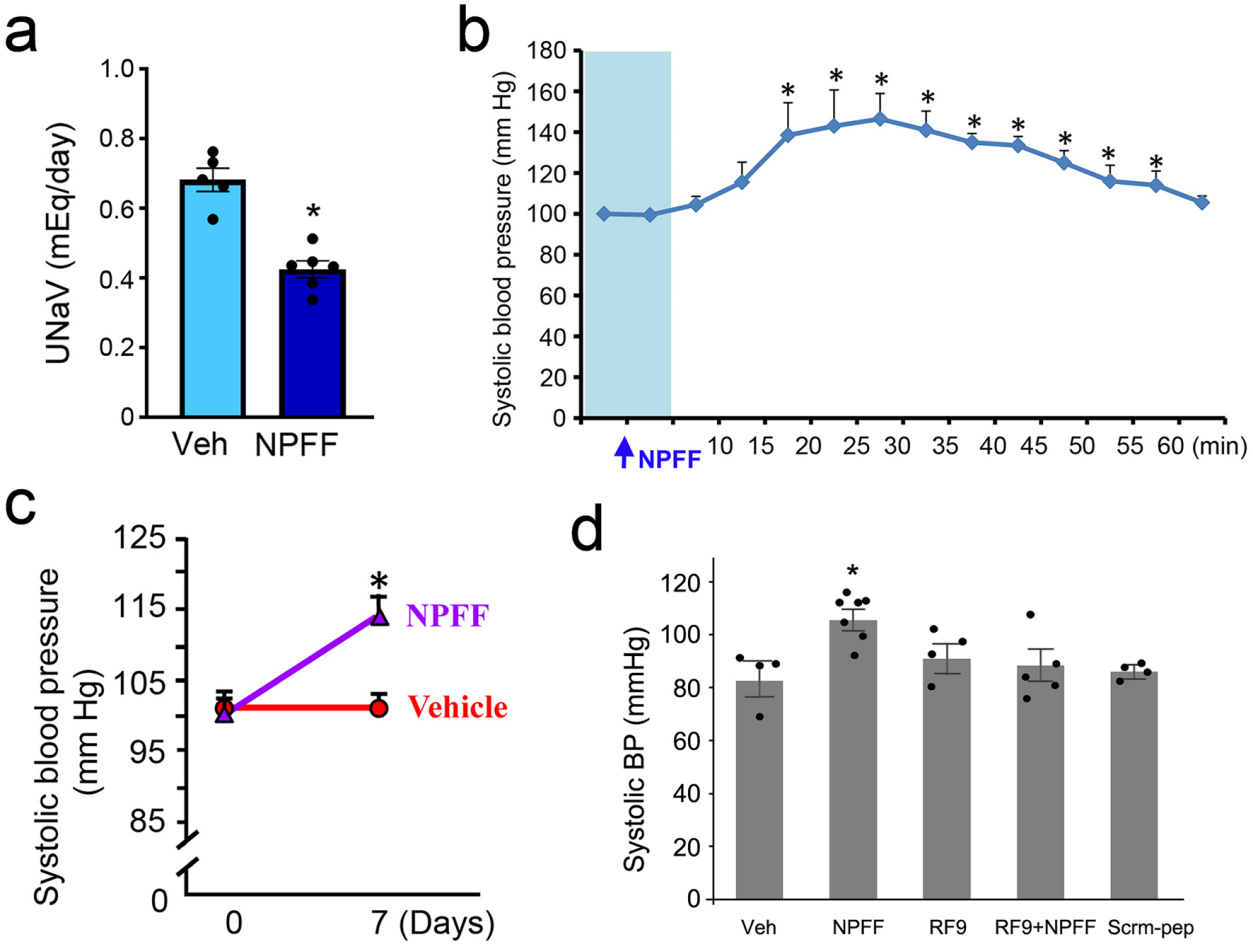
Effect of the renal subcapsular infusion of NPFF on urinary sodium excretion and systolic blood pressure in C57BL/6 mice. (**a**) NPFF (9.25 μmol, 0.5 μL/h), chronically infused underneath the renal capsule, decreased renal sodium excretion in conscious C57BL/6 mice fed a normal salt diet. UNaV, urinary sodium excretion. n = 5–6, * *p* < 0.05, Student’s *t* test. (**b**) Acute effect of NPFF on systolic blood pressure, measured from the aorta, via the carotid artery, caused by a single-dose injection (10 µg in 100 µL) of NPFF underneath the renal capsule. n = 4, * *p* < 0.05 versus basal, one-way ANOVA, Holm-Sidak post-hoc test. (**c**) Chronic effect of NPFF on systolic blood pressure, measured by carotid artery in conscious mice with chronic renal subcapsular infusion of NPFF (9.25 μmol, 0.5 μL/h) for seven days. n = 4, * *p* < 0.05 versus vehicle (0 day), one-way ANOVA, Holm-Sidak post-hoc test. (**d**) Acute effect of NPFF on systolic blood pressure (measured by tail cuff) caused by a single renal subcapsular injection of vehicle (100 µL saline), NPFF (10 µg/100 µL), RF9 (10 µg/100 µL), RF9 + NPFF (10 µg each/100 µL), or scrambled peptide (Scrm-pep, 10 µg/100 µL) in pentobarbital-anesthetized mice. n = 4–7, * *p* < 0.05 versus vehicle (saline, 0.9% NaCl), RF9, NPFF-R1 and NPFF-R2 antagonist; Scrm-pep, NPFF scrambled peptide; one-way ANOVA, Student–Newman–Keuls test.

Previous studies have shown that renal subcapsular infusion is a practical and reproducible method to study local kidney function^33–35^. To determine the effect of NPFF in the kidney, NPFF was acutely and chronically infused underneath the renal capsule. In vivo, NPFF, chronically infused underneath the renal capsule, decreased renal sodium excretion (from 0.68 ± 0.07 mEq/day, n = 5 to 0.43 ± 0.06 mEq/day, n = 6) in conscious C57BL/6 mice fed a normal salt diet (Fig. 8a). In C57BL/6 mice, a single dose (10 µg in 100 µL) of NPFF, which was rapidly injected underneath the renal capsule, increased blood pressure after 15 min that was sustained for about 1 h (Fig. 8b). The chronic renal subcapsular infusion (as in Fig. 8a) of NPFF (9.25 μmol, 0.5 μL/h) for 7 days also increased the blood pressure (114.5 ± 5.0 mmHg, n = 4); the infusion of vehicle (saline) did not affect the blood pressure (96.4 ± 3.0 mmHg, n = 4) (Fig. 8c).

To study further whether the NPFF-mediated increase in blood pressure was through its receptors, blood pressure was measured by tail cuff with the acute administration of RF-9 in conscious mice (Fig. 8d). Consistent with the carotid artery measurement of blood pressure under anesthesia (Fig. 8b), subcapsular injection of a single dose (10 µg in 100 µL) of NPFF significantly increased systolic BP within 15–35 min after injection compared with that of the vehicle, saline (0.9% NaCl, 100 µL) (NPFF: 105.5 ± 3.44 mmHg, Vehicle: 82.6 ± 5.65 mmHg). RF-9 (10 µg in 100 µL) prevented the NPFF-mediated increase in BP (88.5 ± 5.90 mmHg), whereas RF-9 alone had no effect (90.9 ± 4.70 mmHg) (Fig. 8d). Of note, scrambled peptide (10 µg in 100 µL) also had no effect on BP (86.1 ± 1.83 mmHg) (Fig. 8d).

## Discussion

NPFF, like dopamine, was originally found in the brain, but subsequent studies showed its synthesis in peripheral organs^9–12,26–28^. The current study demonstrated that NPFF and its receptors are expressed in the brain and the kidney. NPFF is an endogenous neuropeptide that is predominantly present in the mammalian CNS and is implicated in pain modulation by regulating opioid signaling; other physiological functions are also affected, including the regulation of blood pressure^15^. The distribution and expression of NPFF and its receptors in the CNS have been studied in rats, mice, and humans^10–12,26–28,36–39^. Our data also revealed extensive distribution of NPFF-R1 and NPFF-R2 in the brain, particularly in the hypothalamus, including OVLT, SON, ARC, PVN, and HP. NPFF-R1 had relatively greater expression in ARC than PVN, while NPFF-R2 had similar strong expression in both ARC and PVN and other hypothalamic areas. The distribution of NPFF receptors in the brain is consistent with previous reports in mice^10^. In rats, strong *Npff-r1* expression is observed in the lateral septum, PVN, VMH, DMH, HP, thalamus, amygdala, olfactory bulb, and medulla oblongata; low to moderate expression is observed in the dorsal motor nucleus of the vagus, substantia nigra, and locus coeruleus^11,28,36,39^. *Npff-r2* is highly expressed in the hypothalamus, medulla oblongata, piriform cortex, lateral parabrachial nucleus, thalamus, lateral lemniscus, and trigeminal nucleus^11,28,36,39^. The expression of *Npff* receptors in dopaminergic, NPY, and other neuroendocrine-related neurons in PVN and ARC of hypothalamus areas^15,38–40^ may explain the involvement of *Npff* receptors in central blood pressure regulation, which warrants further investigation.

The expression and distribution of NPFF-R1 and NPFF-R2 in peripheral tissues, especially in the kidney, are not fully known. Limited mRNA expression of *Npff* is observed in the pancreas, lung, spleen, heart, adrenal gland, and skin^11,12,26,39^. The pancreas and adipose tissues have a considerable *Npff* expression in mice^41^, consistent with its role in the regulation of glucose and lipid metabolism^41,42^. Detectable levels of *Npff -r1* mRNA expression are also evident in the adrenal gland, eye, intestine, kidney, lung, ovary, and spleen^11,12,28^. By contrast, *Npff -r2* mRNA is highly expressed in adipose tissue, heart, kidney, retina, salivary gland, stomach, and urinary bladder^11,12,39^. Recent single-cell RNA sequencing showed considerable *NPFF-R1* and detectable *NPFF-R2* expression in hRPTCs (www.proteinatlas.org). However, considerable discrepancies in NPFF receptor expression exist in studies in CNS and peripheral tissues that may be due to the use of different methods and species ^9,11,12,26,28,37,39^.

NPFF receptors are speculated to be expressed highly on the plasma membrane because they are G protein-coupled receptors. However, we found that NPFF-R1 and NPFF-R2 were distributed in both plasma membrane and cytoplasm in human and mouse RPTCs. The predominant cytoplasmic distribution of NPFF-R1 and NPFF-R2 is consistent with that observed in gonadotropin-releasing hormone neurons^43^ and epididymal white adipose cells^42^. Of note, the few NPFF receptors that overlapped with DAPI in our immunofluorescence study could indicate their potential nuclear localization, which needs further investigation.

The transcripts and proteins of NPFF and its receptors are expressed in human and mouse RPTCs, enabling NPFF to function as an autocrine system in the kidney, similar to the renal dopaminergic system^4–6^. The NPFF serum concentration in this study is similar to the NPFF plasma levels in mice and nonobese humans^41^ but higher than that found in an earlier study in healthy humans^44^. The lower plasma NPFF concentrations could be caused by the use of frozen pooled plasma^44^, because NPFF is extremely sensitive to freeze–thaw cycles^41^. The circulating NPFF contains NPFF released from the spinal cord, the pancreas, and possibly the kidney or other unknown tissues^10,11,15,39,41,44^. Our study is the first report on kidney NPFF concentrations and renal NPFF signaling regulates blood pressure in mice. NPFF primarily exerts its functions via the Gαi/o protein which inhibits adenylate cyclase^13^, while NPFF-R2 can also couple to Gαs protein which leads to the stimulation of adenylate cyclase activity^14^. In mRPTCs, the observation that NPFF, RFRP-3, and AC-263093 attenuated forskolin-stimulated cAMP production corroborates the preferential linkage of these two NPFF receptors to Gαi/o. The time- and concentration-dependent inhibition of forskolin-induced cAMP production by NPFF further confirms a functional autocrine model of the NPFF system in RPTCs.

In hypertension, sympathetic activity is increased whereas parasympathetic activity is decreased within the autonomic nervous centers in the hypothalamus and the brainstem^15^. Inhibition of adrenergic activity attenuates the increase in blood pressure caused by NPFF injection in the brainstem^16^, indicating a role of NPFF in the increase in sympathetic activity. NPFF levels are decreased in the DMV of human hypertensive subjects^23^ and spontaneously hypertensive rats^45^, leading to a decrease in vagal activity and baroreflex dysfunction. The renal NPFF system could regulate blood pressure in the physiological state because physiological concentrations of NPFF (pM) decreased forskolin-stimulated cAMP production, and RF-9, the NPFF-R1 and NPFF-R2 antagonist, reversed both RFRP3- (NPFF-R1 agonist) and AC263093- (NPFF-R2 agonist)-mediated decrease in cAMP production in RPTCs. Moreover, the renal subcapsular infusion of physiological concentrations of NPFF also decreased urinary sodium excretion and increased the blood pressure. Whereas scrambled peptide had no effect on the increase in blood pressure, RF-9 prevented the acute NPFF-mediated increase in blood pressure. The current observations are consistent with previous observations of others^18,29,46^. The intravenous administration of NPFF in anesthetized rats increased blood pressure that was attenuated by daY8Fa, an NPFF-R1 and NPFF-R2 antagonist^46^. In anesthetized rats, RF-9 almost completely abrogated the increase in blood pressure caused by the lateral cerebral ventricular injection of NPFF^29^ or the intrathecal administration of NPFF^18^. These results indicate that the effects of NPFF on cAMP production and blood pressure were exerted through its own receptors.

However, the sodium transporters related to the involved NPFF receptors in the regulation of blood pressure in the kidney are not clear at this time. The protein expression of either NHE3 or Na^+^/K^+^-ATPase in *Npff-r1* or *Npff-r2* deficient kidney cortices in C57BL/6 mice fed with a normal salt diet cannot explain the NPFF-mediated increase in blood pressure. The RPT is the site where two-thirds of filtered sodium is reabsorbed; NHE3 is recognized to be the most important mediator of sodium transport across the luminal membrane of this nephron segment^47^. Dopamine has been shown to inhibit NHE3 activity by decreasing its expression and increasing its phosphorylation^48,49^. NPFF and the D1-like receptor agonist, fenoldopam, exerted a counter regulatory effect on cAMP production; NPFF-R1 and NPFF-R2, colocalized and physically interacted with D_1_R in the RPT. NPFF-R1, unlike NPFF-R2, also colocalized and interacted with the D_5_R in the RPT.

The renal dopaminergic system maintains normal blood pressure by increasing sodium excretion, especially in states of moderate sodium excess^4–6,50^. It is possible that the decrease in renal sodium excretion and increase in blood pressure due to the intrarenal administration of NPFF is related, in part, to its counter regulation of the renal dopaminergic system. In the kidney, the dopaminergic system also counter regulates the renin–angiotensin–aldosterone system^4–6,51^. We have also found that the angiotensin type I receptor co-immunoprecipitated with NPFF-R1 and NPFF-R2 in hRPTCs and infusion of NPFF and angiotensin II synergistically increased blood pressure in C57BL/6 mice (Asico LD and Jose PA, unpublished observation). In rats, the peripheral NPFF-induced increase in blood pressure was attenuated by blocking α1-adrenergic receptors^19^, indicating a potential interaction of the NPFF system with the adrenergic system. Therefore, the effect of NPFF on sodium excretion and blood pressure is probably related to the counter-regulation of a renal natriuretic system (dopamine) and/or positive interaction with angiotensin II and  the adrenergic system. NPFF weakly activates the MAS receptor in HEK293 cells^52^ and promotes macrophage M2 polarization and prevents inflammation in adipose tissues^41^. Inflammation participates in the regulation of renal function and blood pressure^1,2,4–6,8^. Similar to Ang II, NPFF has a major effect on the increase in blood pressure, however, NPFF could also activate the MAS receptor and protect against an increase in blood pressure^52^. However, the role of NPFF in these interactions in the regulation of sodium excretion and blood pressure remains to be determined.

In addition to NPFF, a total of five groups of RF-amide peptides have been identified in mammals, including RF-amide related peptides (RFRP), prolactin-releasing peptides, kisspeptin, and pyroglutamylated RF-amide peptides^53^. Each peptide group has specific cognate receptors with considerable cross-reactivity with the receptors of the other groups^11,39,54^. NPFF has an affinity to both NPFF-R1 and NPFF-R2^11,17,39^. RF-9, a dual NPFF-R1 and NPFF-R2 antagonist, has been reported as a Kiss1 receptor agonist in the gonadotropin system^32^, while reduced circulating kisspeptin levels are observed in pre-eclampsia compared with normotensive pregnancy^55^. The acute intrarenal administration of RF-9, alone, had no effect on blood pressure but as aforementioned, its chronic renal subcapsular infusion slightly decreased the blood pressure. In RPTCs, both RFRP-3, an NPFF-R1 agonist, and AC-263093, an NPFF-R2 agonist, were able to inhibit the forskolin-stimulated cAMP production, suggesting that both NPFF-R1 and NPFF-R2 are involved in NPFF’s inhibition of cAMP production. By contrast, RFRP-2, which, by itself, has a low affinity to NPFF receptors, did not affect the cAMP production, highlighting the specificity of NPFF action on NPFF-R1 and NPFF-R2. In the nucleus tractus solitarius, NPFF can bind to the RFRP receptor, which is co-expressed with neuropeptide Y (NPY)^54^. NPY and at least one of its receptors are present in RPTs^53^, whose activation can mediate the increase in blood pressure^56^. The intravenous administration of neuropeptide 26RFa, an agonist of GPR103, increases blood pressure, which can be attenuated by pretreatment with an antagonist of NPY receptor^57^. D1-like receptors can inhibit the vascular smooth muscle proliferation caused by NPY^58^. Therefore, it is important to determine whether NPY contributes to the NPFF-induced cAMP signaling and subsequent blood pressure regulation.

Despite efforts to obtain the most specific commercially available antibodies for the staining of NPFF receptors in both cells and kidney sections, the specificity of their staining still needs further confirmation. This is a particular concern with NPFF-R1 which has a weak staining in RPTCs and brain. Whether or not NPFF may function in the kidney through NPFF-R2 needs to be evaluated in a future study. It should be noted, however, that RNAscope and RT-PCR showed the expressions of *Npff (NPFF)*, *Npff-r1(NPFF-R1)*, and *Npff-r2(NPFF-R2)* mRNA in the RPT.

Recent genome-wide association studies demonstrated that a single nucleotide polymorphism (SNP) of *NPFF*, rs11170566, is associated with migraine, inflammation, and cardiovascular disorders^59^. SNPs of *Npff-r1* may be related to glucose metabolism and growth-related traits of the common carp^60^. SNPs of *Npff-r2* are associated with impaired lipid metabolism, obesity, and inflammation^61,62^. Moreover, epidemiological studies show an association of blood pressure with polymorphisms in the GPR10 receptor^63^, the cognate receptor for prolactin-releasing peptide, which may regulate blood pressure via NPFF-R2^64^. The association of SNPs of *NPFF* and its receptors with inflammation and aberrant metabolism of glucose and lipid is consistent with the notion that hypertension is a chronic inflammatory disorder^65^. Henceforth, studies are needed to determine if the gene variants of NPFF and its receptors can directly influence renal sodium transport and blood pressure.

## Methods

### Antibodies and reagents

Primary antibodies used in this study are listed in the Supplementary Table S1. The D_1_R and D_5_R antibodies have been thoroughly validated^66–69^, using the methods advocated by the ad hoc International Working Group for Antibody Validation^70^. The commercial NPFF-R1 and NPFF-R2 antibodies were characterized by immunoblotting the kidney cortices from mice infused with specific *Npff-r1* and *Npff-r2* siRNA underneath the kidney capsule (Supplementary Fig. S5). Normal mouse (Cat. No. sc-2025), rabbit IgG (Cat. No. 2729), and chicken IgY (Cat. No. AC146) antibodies were purchased from Santa Cruz Biotechnology (Santa Cruz, CA), Cell Signaling Technology (Danvers, MA) and Sigma-Aldrich (St. Louis, MO), respectively. Appropriate secondary Alexa Fluor antibodies were purchased from Thermo Fisher Scientific (Gaithersburg, MD). Fenoldopam (Cat. No. 1659), NPFF (Cat. No. 3137), and RF-9 (Cat. No. 3672) were purchased from Tocris (Minneapolis, MN). RFRP-2 (Cat. No. 048-44) and RFRP-3 (Cat. No. 048-46) were purchased from Phoenix Pharmaceuticals (Burlingame, CA). The scrambled peptide was synthesized by GenScript (Piscataway, NJ). AC-263093 (Cat. No. orb611290) was purchased from Biorbyt (St. Louis, MO). Forskolin (Cat. No. F3917) and other reagents were purchased from Sigma (St. Louis, MO).

### Cell culture

hRPTCs^67–69,71^ were verified of their RPTC origin by the expression of γ-glutamyl transpeptidase and NHE3, as previously reported^67,68^. mRPTCs, kindly supplied by Dr. Ulrich Hopfer (Case Western Reserve University School of Medicine), were isolated from C57BL/6 mice and characterized, as previously described^72^. Immortalized RPTCs with low passages were cultured in a 1:1 mixture of DMEM and Ham’s F-12 medium, supplemented with 5% fetal bovine serum, selenium (5 ng/mL), insulin (5 μg/mL), transferrin (5 μg/mL), hydrocortisone (36 ng/mL), triiodothyronine (4 pg/mL), and epidermal growth factor (10 ng/mL).

### RT-PCR

Reverse transcriptase-polymerase chain reaction (RT-PCR) was performed, as previously described^72^. Briefly, RNA of hRPTCs was extracted with Trizol (Invitrogen, Carlsbad, CA) and further purified, using the RNeasy RNA Extraction Mini kit (Qiagen). RNA samples were converted into first-strand cDNA using an RT2 First Strand kit (SABiosciences-Qiagen). The transgenes were amplified (Taq DNA polymerase, Invitrogen) with specific NPFF, NPFF-R1, and NPFF-R2 primer pairs (Supplementary Table S2) at 95 °C for 3 min, followed by 35 cycles at 94 °C for 30 s, 53 °C for 30 s, 72 °C for 45 s, and 60 °C for 10 min. The PCR products of NPFF, NPFF-R1, and NPFF-R2 were resolved in 1.5% agarose gel in Tris/Borate/EDTA buffer, containing 0.5 µg/mL ethidium bromide and subjected to sodium dodecyl sulfate–polyacrylamide gel electrophoresis (SDS-PAGE). The gels were photographed under ultraviolet light.

### Western blot

Western blotting was performed as previously described^69,71^. Briefly, kidney cortices were lysed in 1 × RIPA lysis buffer (Millipore, Billerica, MA), containing protease and phosphatase inhibitor cocktail (Thermo Fisher Scientific, Rockford, IL), and the samples were adjusted to have the same protein concentration. The proteins were separated by SDS-PAGE, transferred onto nitrocellulose membranes, and then probed with primary antibodies and appropriately conjugated secondary antibodies. The images were visualized by a LiCor Odyssey Imaging system.

### Immunofluorescence imaging

Immunofluorescence imaging was performed, as previously described^69,71,74^. HRPTCs or mRPTCs were grown on poly-d-lysine-coated coverslips and fixed with 4% paraformaldehyde in phosphate-buffered saline (PBS) for 20 min at room temperature. After washing with PBS, the cells, fixed on coverslips, were incubated with primary anti-NPFF, anti-NPFF-R1, anti-NPFF-R2, anti-D_1_R, or anti-D_5_R antibodies overnight at 4 °C. The coverslips were then incubated with the proper Alexa Fluor-488 and -555 secondary antibodies for 2 h at 4 °C. The coverslips were mounted in a proper antifade mounting medium and sealed onto glass slides.

Human kidney sections (Imgenex, San Diego, CA, USA) were prepared for antigen retrieval, using heat and pressure and immunostained for NPFF-R1, NPFF-R2, D_1_R, and D_5_R antibodies. Wheat germ agglutinin, conjugated with Alexa Fluor 647, was used to target the lectin-rich brush border and plasma membranes of RPTs. DAPI was used to visualize nuclei. For negative controls, the primary antibodies were replaced with normal rabbit serum at the appropriate dilutions.

Mouse brains were coronally sectioned at 10 μm thickness using a cryostat, and the sections, including the entire forebrain regions underwent standard immunohistochemistry, as previously reported^73^ to study brain distribution of NPFF-R1 and NPFF-R2. For NPFF-R1 and NPFF-R2 immunostaining, the primary antibodies were diluted 1:100 in blocking buffer (3% BSA and 0.3% Triton X-100 in PBS) and the slides were incubated for 2 days in a cold room. Alexa Fluor 488-conjugated secondary antibody was diluted 1:1000 in blocking buffer and the slides were incubated for 2 h at room temperature to visualize both immunoreactive signals. The slides were cover-slipped and subjected to microscopy, using a BX43F Olympus fluorescent microscope (Center Valley, PA) with a DP80 camera. Preliminary experiments to determine the appropriate working concentration and incubation time for each antibody were performed. For negative controls, the primary antibodies were removed, and the slides were only incubated with the blocking buffer followed by Alexa Fluor 488-conjugated secondary antibody for the appropriate time period.

### Cyclic AMP assay

Cyclic AMP (cAMP) was assayed using a direct immunoassay kit (Arbor Assays, Ann Arbor, MI), as previously described^69,71^. Briefly, hRPTCs and mRPTCs were grown in 12-well plates. The RPTCs at ~ 75% confluence were pretreated with the phosphodiesterase inhibitor 3-isobutyl-1-methylxanthine (IBMX; 1 M; Sigma-Aldrich, St. Louis, MO, USA), before the addition of a D1-like receptor agonist, fenoldopam (1 µM/30 min), NPFF, RFRP-2, RFRP-3, and/or AC-263093, at the indicated concentrations and time, and re-challenged with forskolin (10 µM) or PBS for 30 min. The cell lysates were prepared to determine the protein concentration, using the BCA protein assay kit (Thermo Scientific, Rockford, IL, USA). After quantification, the same amount of cell lysates (Supplementary Fig. S3) or culture supernatants (Figs. 3 and 6) were used to determine cAMP concentrations with reading the optical density at 450 nm by an ELISA plate reader.

### Co-immunoprecipitation assay

Co-immunoprecipitation was performed using a Dynabeads kit (Thermo Fisher Scientific), as previously described^74^. Briefly, ~ 90% confluent hRPTCs were harvested, and the cell pellets were lysed in a lysis buffer (20 mM Tris·HCl, pH 8.0/1 mM EDTA/1 mM NaN3 /2 mM DTT/0.25 M sucrose), with 0.2 mM phenylmethylsulfonyl fluoride, and protease and phosphatase inhibitor cocktail. Five µg of anti-D_1_R, anti-D_5_R, anti-NPFF-R1, or anti-NPFF-R2 antibodies were conjugated with Dynabeads in 0.5 mL of slurry. The cell lysates were then incubated with the conjugated antibodies at 4 °C for 4 h, followed by proper washing. The controls were normal rabbit IgG and chicken IgY. Proteins bound to the beads were eluted in 60 µL of loading buffer at 65 °C for 15 min, separated by 10% SDS-PAGE, and transferred onto a nitrocellulose membrane for incubation with the detecting antibody, followed by the appropriate secondary antibody, before visualization with a LiCor Odyssey Imaging system.

### Targeted quantification of NPFF with Liquid chromatography- tandem mass spectrometry (LC–MS/MS)

Targeted LC–MS/MS method was used to measure NPFF in the mouse serum and kidney as previously described^75,76^. Briefly, C57BL/6 mouse serum and kidney samples were homogenized and sonicated in ice-cold acidified 90% methanol buffer to precipitate large proteins and extract NPFF in the supernatant. Supernatant samples were collected after centrifugation at 18,000×*g* for 30 min at 4 °C. Molecular weight cutoff (MWCO, 10 K) ultra-centrifugal filter (Sigma) was used to enrich molecules lower than 10 K MW, which were then dried down and desalted by Waters HLB solid phase extraction plate. ^13^C_5_, ^15^N folic acid was spiked into the sample as the internal standard (I.S.). LC–MS–MS analysis was conducted using a Dionex Ultimate 3000 RSLCnano system coupled with a Thermo Scientific Q-Exactive HFX mass spectrometer as described previously^75^. An Easy-spray PepMap C18 LC column (2 μM, 100 Å, 75 μM × 15 cm) was used to separate peptide samples with a 1 h LC gradient. Serial concentrations of NPFF standards (Cayman) were spiked into a highly diluted sample matrix (no detectable NPFF signal) with I.S. to generate calibration curves. A parallel reaction monitoring (PRM) method was established by using the a2, b2, y4, y6 fragment ions from NPFF peptide, normalized to the fragment ion from the I.S. Data analysis was conducted with Skyline software^76^ and R studio.

### In situ RNA hybridization by RNAScope

In situ RNA hybridization was performed using RNAscope technology (Advanced Cell Diagnostics, Newark, CA), as previously described^72^. Briefly, thin sections (5 µm) of formalin-fixed, paraffin-embedded mouse kidneys were deparaffinized in xylene and rehydrated with step-down concentrations of ethanol. The tissues were then treated serially with the following: 10-min immersion in pretreatment 1 solution (endogenous H_2_O_2_ blocker); 100 °C, 15-min immersion in pretreatment 2 solution; and protease digestion, 40 °C for 10 min. The tissues were rinsed with water after each pretreatment step and then hybridized with specific *Npff*, *Npff-r1*, and *Npff-r2* RNAscope probes at 40 °C for 2 h (Advanced Cell Diagnostics). The specific probes were targeted for mice: *Npff* mRNA, Mm-Npff1 (NM_018787.1., Cat. No. 479901) with region designed against 117–313 nt; *Npff-r1* mRNA, Mm-Npff1 (NM_001177511.1., Cat. No. 410161) with region designed against 14–1298 nt; *Npff-r2* mRNA, and Mm-Npff2 (NM_133192.3., Cat. No. 410171) with region designed against 233–1342 nt. Mm‐PPIB, *Mus musculus* peptidylprolyl isomerase B (Ppib, Cat. No. 313911) was the positive control. *Bacillus subtilis* dihydrodipicolinate reductase (dapB, Cat. No. 310043) was the negative control. After the wash and buffer steps, the signal was amplified, using a multistep process (Each RNAscope 2.5 HD Reagent Kit—BROWN, Cat. No. 322300; HybEZ Hybridization System, Cat. No. 310010). Horseradish peroxidase (HRP)-labeled probes were visualized by the application of 3, 3′-diaminobenzidine (DAB). The sections were then counterstained with hematoxylin.

### Urinary sodium excretion and blood pressure measurement

Adult C57BL/6 mice (male, 8-week-old), purchased from Jackson Laboratory (Bar Harbor, ME), were housed in a temperature-controlled facility with a 12:12-h light–dark cycle and fed with regular mouse chow and water ad libitum for at least 2 weeks before any studies were performed. Renal *Npff-r1* and *Npff-r2* were silenced by the chronic renal subcapsular infusion of specific *Npff-r1* and *Npff-r2* siRNA (Cat. No. SI01037379 and SI04925039 respectively, Qiagen, Germantown, MD), via an osmotic minipump, as previously described^69,71,72^. Briefly, the mice were uninephrectomized 1 week prior to the implantation of the minipump. For the minipump implantation, the mice were anesthetized with pentobarbital (50 mg/kg body weight, intraperitoneally). The osmotic minipumps (100 µL; flow rate: 0.5 µl/h) were filled with validated *Npff-r1*-specific siRNA, *Npff-r2*-specific siRNA (Cat. No. SI01037379, Cat. No. SI04925039, Qiagen), or non-silencing mock siRNA (Cat. No. 03650318, Qiagen), as control. The siRNAs were dissolved in an in vivo transfection reagent (TransIT In Vivo Gene Delivery System, Mirus), under sterile conditions. The minipumps were fitted with a polyethylene delivery tubing (Alzet #0,007,701) and the tip of the tubing was inserted within the subcapsular space of the remaining kidney. The efficiency of siRNA infusion was analyzed by real-time PCR, performed on an Applied Biosystems® ViiA™ 7 Real-Time PCR System (Foster City, CA). The primers (SABiosciences-Qiagen) used for qRT-PCR are in Supplementary Table S3. The data were analyzed using the ΔCt method^68,72^, the gene and protein expressions of *Npff-r1* or *Npff-r2* after the 7-days siRNA infusion are shown in Supplementary Figure S6 and Supplementary Figure S5, respectively.

Twenty-four urine samples were collected from mice individually housed in metabolic cages. The mice were acclimatized in the metabolic cages for 24 h before the collection of urine. Urine sodium concentration was measured using Easylyte Analyzer (Medica Corporation, Bedford, MA). Urinary sodium excretion (UNaV) was calculated as urine volume × sodium (mEq/liter).

Blood pressures of mice with acute renal subcapsular infusion of NPFF were measured (Cardiomax II; Columbus Instruments, Columbus OH) from the aorta via the carotid artery under pentobarbital sodium anesthesia (50 mg/kg)^71,72^ and by tail cuff^69^ with a CODA system (Kent scientific corporation, Torrington, CT, USA) in conscious mice as described previously.

Blood pressure of chronic NPFF (9.25 μmol, 0.5 μL/h, 0.05 nmol/day) and saline (0.9%NaCl) infusion groups was recorded by telemetry in conscious mice, as previously described^35,72^.

The studies were conducted by following the guidelines set by the US National Institutes of Health for the ethical treatment and handling of animals in research and approved by the Institutional Animal Care and Use Committee (IACUC) of The George Washington University. All experiments were performed in accordance with relevant guidelines and regulations and the recommendations in the ARRIVE guidelines.

### Statistical analysis

Data are presented as mean ± standard deviation (SD). All the cell experiments were performed using a minimum of triplicate wells and repeated at least twice, which is our laboratory routine for cAMP assays. However, the inter- and intra-assay variability was not assessed in the LC–MS/MS NPFF quantification.

Differences between two groups were assessed by Student’s t-test and differences among three or more groups were assessed by one-way ANOVA with the Newman-Keuls or Holm-Sidak test. *P* values < 0.05 were considered statistically significant (SigmaPlot, San Jose, CA).

### Ethics approval and consent to participate

The mouse experiments were performed according to a protocol (A353) approved on December 8, 2017, and protocol A2022-014 that is good until March 23, 2025) by the George Washington University Institutional Animal Care and Use Committee. The use of hRPTCs followed a protocol, HSR#13310, approved by the University of Virginia Institutional Review Board, which is renewed annually.

### Supplementary Information

Supplementary Information.

## Data Availability

The data are available from the corresponding author upon reasonable request.

## References

[1] Harrison DG, Coffman TM, Wilcox CS (2021). Pathophysiology of hypertension: The mosaic theory and beyond. Circ. Res..

[2] Rucker AJ, Rudemiller NP, Crowley SD (2018). Salt, hypertension, and immunity. Annu. Rev. Physiol..

[3] Seidel E, Scholl UI (2017). Genetic mechanisms of human hypertension and their implications for blood pressure physiology. Physiol. Genom..

[4] Jose PA, Eisner GM, Felder RA (1998). Renal dopamine receptors in health and hypertension. Pharmacol. Ther..

[5] Banday AA, Lokhandwala MF (2008). Dopamine receptors and hypertension. Curr. Hypertens. Rep..

[6] Harris RC, Zhang MZ (2012). Dopamine, the kidney, and hypertension. Curr. Hypertens. Rep..

[7] Albrecht FE (1996). Role of the D1A dopamine receptor in the pathogenesis of genetic hypertension. J. Clin. Invest..

[8] Hollon TR (2002). Mice lacking D5 dopamine receptors have increased sympathetic tone and are hypertensive. J. Neurosci..

[9] Yang HY, Fratta W, Majane EA, Costa E (1985). Isolation, sequencing, synthesis, and pharmacological characterization of two brain neuropeptides that modulate the action of morphine. Proc. Natl. Acad. Sci. U.S.A..

[10] Gouardères C, Puget A, Zajac JM (2004). Detailed distribution of neuropeptide FF receptors (NPFF1 and NPFF2) in the rat, mouse, octodon, rabbit, guinea pig, and marmoset monkey brains: A comparative autoradiographic study. Synapse.

[11] Bonini JA (2000). Identification and characterization of two G protein-coupled receptors for neuropeptide FF. J. Biol. Chem..

[12] Elshourbagy NA (2000). Receptor for the pain modulatory neuropeptides FF and AF is an orphan G protein-coupled receptor. J. Biol. Chem..

[13] Mollereau C (2002). Pharmacological characterization of human NPFF(1) and NPFF(2) receptors expressed in CHO cells by using NPY Y(1) receptor antagonists. Eur. J. Pharmacol..

[14] Gherardi N, Zajac JM (1997). Neuropeptide FF receptors of mouse olfactory bulb: Binding properties and stimulation of adenylate cyclase activity. Peptides.

[15] Jhamandas JH, Goncharuk V (2013). Role of neuropeptide FF in central cardiovascular and neuroendocrine regulation. Front. Endocrinol..

[16] Jhamandas JH, Mactavish D (2002). Central administration of neuropeptide FF (NPFF) causes increased neuronal activation and up-regulation of NPFF gene expression in the rat brainstem. J. Comp. Neurol..

[17] Laguzzi R, Nosjean A, Mazarguil H, Allard M (1996). Cardiovascular effects induced by the stimulation of neuropeptide FF receptors in the dorsal vagal complex: An autoradiographic and pharmacological study in the rat. Brain Res..

[18] Fang Q, Li N, Jiang TN, Liu Q, Li YL, Wang R (2010). Pressor and tachycardic responses to intrathecal administration of neuropeptide FF in anesthetized rats. Peptides.

[19] Allard M, Labrouche S, Nosjean A, Laguzzi R (1995). Mechanisms underlying the cardiovascular responses to peripheral administration of NPFF in the rat. J. Pharmacol. Exp. Ther..

[20] Zhang M (2020). Synthesis and biological characterization of cyclic disulfide-containing peptide analogs of the multifunctional opioid/neuropeptide FF receptor agonists that produce long-lasting and nontolerant antinociception. J. Med. Chem..

[21] Wojciechowski P, Andrzejewski K, Kaczyńska K (2020). Intracerebroventricular neuropeptide FF diminishes the number of apneas and cardiovascular effects produced by opioid receptors’ activation. Int. J. Mol. Sci..

[22] Thiemermann C, Al-Damluji S, Hecker M, Vane JR (1991). FMRF-amide and L-Arg-L-Phe increase blood pressure and heart rate in the anaesthetised rat by central stimulation of the sympathetic nervous system. Biochem. Biophys. Res. Commun..

[23] Goncharuk VD, Buijs RM, Jhamandas JH, Swaab DF (2014). The hypothalamic neuropeptide FF network is impaired in hypertensive patients. Brain Behav..

[24] Zeng C, Armando I, Yang J, Jose PA (2023). Dopamine receptor D1R and D3R and GRK4 interaction in hypertension. Yale J. Biol. Med..

[25] Moore SC, Vaz de Castro PAS, Yaqub D, Jose PA, Armando I (2023). Anti-inflammatory effects of peripheral dopamine. Int. J. Mol. Sci..

[26] Nieminen ML, Brandt A, Pietilä P, Panula P (2000). Expression of mammalian RF-amide peptides neuropeptide FF (NPFF), prolactin-releasing peptide (PrRP) and the PrRP receptor in the peripheral tissues of the rat. Peptides.

[27] Sun Y, Kuang Y, Zuo Z (2021). Transcriptomic changes in mouse bone marrow-derived macrophages exposed to neuropeptide FF. Genes.

[28] Hinuma S (2000). New neuropeptides containing carboxy-terminal RFamide and their receptor in mammals. Nat. Cell Biol..

[29] Simonin F (2006). RF9, a potent and selective neuropeptide FF receptor antagonist, prevents opioid-induced tolerance associated with hyperalgesia. Proc. Natl. Acad. Sci. U.S.A..

[30] Gouardères C (2007). Functional differences between NPFF1 and NPFF2 receptor coupling: High intrinsic activities of RFamide-related peptides on stimulation of ^[35S^^]^GTPγS binding. Neuropharmacology.

[31] Lameh J (2010). Neuropeptide FF receptors have opposing modulatory effects on nociception. J. Pharmacol. Exp. Ther..

[32] Min L (2015). RF9 acts as a KISS1R agonist in vivo and in vitro. Endocrinology.

[33] Hansen PB, Hashimoto S, Oppermann M, Huang Y, Briggs JP, Schnermann J (2005). Vasoconstrictor and vasodilator effects of adenosine in the mouse kidney due to preferential activation of A1 or A2 adenosine receptors. J. Pharmacol. Exp. Ther..

[34] Konkalmatt PR (2016). Renal rescue of dopamine D2 receptor function reverses renal injury and high blood pressure. JCI Insight.

[35] Tiu AC, Yang J, Asico LD, Konkalmatt P, Zheng X, Cuevas S, Wang X, Lee H, Mazhar M, Felder RA, Jose PA, Villar VAM (2020). Lipid rafts are required for effective renal D1 dopamine receptor function. FASEB J..

[36] Liu Q (2001). Identification and characterization of novel mammalian neuropeptide FF-like peptides that attenuate morphine-induced antinociception. J. Biol. Chem..

[37] Zhang L (2018). Diet-induced adaptive thermogenesis requires neuropeptide FF receptor-2 signalling. Nat. Commun..

[38] Higo S, Kanaya M, Ozawa H (2021). Expression analysis of neuropeptide FF receptors on neuroendocrine-related neurons in the rat brain using highly sensitive in situ hybridization. Histochem. Cell Biol..

[39] Koller J, Herzog H, Zhang L (2021). The distribution of Neuropeptide FF and Neuropeptide VF in central and peripheral tissues and their role in energy homeostasis control. Neuropeptides.

[40] Aquino NSS (2019). Kisspeptin stimulation of prolactin secretion requires Kiss1 receptor but not in tuberoinfundibular dopaminergic neurons. Endocrinology.

[41] Waqas SFH (2017). Neuropeptide FF increases M2 activation and self-renewal of adipose tissue macrophages. J. Clin. Invest..

[42] Alexopoulou F (2022). Lipidated PrRP31 metabolites are long acting dual GPR10 and NPFF2 receptor agonists with potent body weight lowering effect. Sci. Rep..

[43] Constantin S (2021). An inhibitory circuit from brainstem to GnRH neurons in male mice: A new role for the RFRP receptor. Endocrinology.

[44] Sundblom DM, Hyrkkö A, Fyhrquist F (1998). Pulsatile secretion of neuropeptide FF into human blood. Peptides.

[45] Yilmaz A, Kalsbeek A, Buijs RM (2021). Early changes of immunoreactivity to orexin in hypothalamus and to RFamide peptides in brainstem during the development of hypertension. Neurosci. Lett..

[46] Prokai L, Zharikova AD, Juhasz A, Prokai-Tatrai K (2006). Cardiovascular effects of neuropeptide FF antagonists. Peptides.

[47] Zhuo JL, Soleimani M, Li XC (2021). New insights into the critical importance of intratubular Na^+^/H^+^ exchanger 3 and its potential therapeutic implications in hypertension. Curr. Hypertens. Rep..

[48] Hu MC, Di Sole F, Zhang J, McLeroy P, Moe OW (2013). Chronic regulation of the renal Na^+^/H^+^ exchanger NHE3 by dopamine: Translational and posttranslational mechanisms. Am. J. Physiol. Renal Physiol..

[49] Li XX (2001). D(1) dopamine receptor regulation of NHE3 during development in spontaneously hypertensive rats. Am. J. Physiol. Regul. Integr. Comp. Physiol..

[50] Hansell P, Fasching A (1991). The effect of dopamine receptor blockade on natriuresis is dependent on the degree of hypervolemia. Kidney Int..

[51] Li H (2008). Dopamine 5 receptor mediates Ang II type 1 receptor degradation via a ubiquitin-proteasome pathway in mice and human cells. J. Clin. Invest..

[52] Tirupula KC, Desnoyer R, Speth RC, Karnik SS (2014). Atypical signaling and functional desensitization response of MAS receptor to peptide ligands. PLoS One.

[53] Sandvik GK, Hodne K, Haug TM, Okubo K, Weltzien FA (2014). RFamide peptides in early vertebrate development. Front. Endocrinol..

[54] Haefliger JA (1999). Cellular localization, expression and regulation of neuropeptide Y in kidneys of hypertensive rats. Regul. Pept..

[55] Tsoutsouki J, Patel B, Comninos AN, Dhillo WS, Abbara A (2022). Kisspeptin in the prediction of pregnancy complications. Front. Endocrinol..

[56] Winaver J, Abassi Z (2006). Role of neuropeptide Y in the regulation of kidney function. EXS..

[57] Takayasu S (2006). A neuropeptide ligand of the G protein-coupled receptor GPR103 regulates feeding, behavioral arousal, and blood pressure in mice. Proc. Natl. Acad. Sci. U.S.A..

[58] Zhou Y (2015). Inhibitory effect of D1-like dopamine receptors on neuropeptide Y-induced proliferation in vascular smooth muscle cells. Hypertens. Res..

[59] Zhao H (2016). Gene-based pleiotropy across migraine with aura and migraine without aura patient groups. Cephalalgia.

[60] Peng W (2016). An ultrahigh density linkage map and QTL mapping for sex and growth-related traits of common carp (*Cyprinus carpio*). Sci. Rep..

[61] Dahlman I (2007). A common haplotype in the G-protein-coupled receptor gene GPR74 is associated with leanness and increased lipolysis. Am. J. Hum. Genet..

[62] Sahana G (2014). Genome-wide association study using high-density single nucleotide polymorphism arrays and whole-genome sequences for clinical mastitis traits in dairy cattle. J. Dairy Sci..

[63] Bhattacharyya S (2003). Association of polymorphisms in GPR10, the gene encoding the prolactin-releasing peptide receptor with blood pressure, but not obesity in a U.K. Caucasian population. Diabetes.

[64] Ma L (2009). Prolactin-releasing peptide effects in the rat brain are mediated through the Neuropeptide FF receptor. Eur. J. Neurosci..

[65] Mouton AJ, Li X, Hall ME, Hall JE (2020). Obesity, hypertension, and cardiac dysfunction: Novel roles of immunometabolism in macrophage activation and inflammation. Circ. Res..

[66] Stojanovic T (2017). Validation of dopamine receptor DRD1 and DRD2 antibodies using receptor deficient mice. Amino Acids.

[67] Sanada H (1999). Dopamine-1 receptor coupling defect in renal proximal tubule cells in hypertension. Hypertension.

[68] Lee H (2021). Dopamine D5 receptor-mediated decreases in mitochondrial reactive oxygen species production are cAMP and autophagy dependent. Hypertens. Res..

[69] Villar VA (2013). Novel role of sorting nexin 5 in renal D(1) dopamine receptor trafficking and function: Implications for hypertension. FASEB J..

[70] Uhlen M (2021). Response to: Should we ignore western blots when selecting antibodies for other applications?. Nat. Methods.

[71] Villar VA (2013). Sorting nexin 1 loss results in D5 dopamine receptor dysfunction in human renal proximal tubule cells and hypertension in mice. J. Biol. Chem..

[72] Jiang X (2017). Gastrin stimulates renal dopamine production by increasing the renal tubular uptake of l-DOPA. Am. J. Physiol. Endocrinol. Metab..

[73] Jeong JK, Horwath JA, Simonyan H, Blackmore KA, Butler SD, Young CN (2019). Subfornical organ insulin receptors tonically modulate cardiovascular and metabolic function. Physiol. Genom..

[74] Li H, Li HF, Felder RA, Periasamy A, Jose PA (2008). Rab4 and Rab11 coordinately regulate the recycling of angiotensin II type I receptor as demonstrated by fluorescence resonance energy transfer microscopy. J. Biomed. Opt..

[75] Li H, Uittenbogaard M, Navarro R, Ahmed M, Gropman A, Chiaramello A, Hao L (2022). Integrated proteomic and metabolomic analyses of the mitochondrial neurodegenerative disease MELAS. Mol. Omics.

[76] Pino LK, Searle BC, Bollinger JG, Nunn B, MacLean B, MacCoss MJ (2020). The Skyline ecosystem: Informatics for quantitative mass spectrometry proteomics. Mass Spectrom. Rev..

