# Supplementary material for: Renal autocrine neuropeptide FF (NPFF) signaling regulates blood pressure
Source: Sci Rep. 2024 Jul 4;14:15407. doi: 10.1038/s41598-024-64484-9 (PMC11224344; doi:10.1038/s41598-024-64484-9)

**SUPPLEMENTARY MATERIALS**

Hewang Lee*^1,2,#^, Bibhas Amatya^1,#^, [Van Anthony M. Villar](https://pubmed.ncbi.nlm.nih.gov/?sort=date&size=200&term=Villar+VA&cauthor_id=19380616)^1,2†^, [Laureano D. Asico](https://pubmed.ncbi.nlm.nih.gov/?sort=date&size=200&term=Asico+LD&cauthor_id=29080975)^1,2^, Jin Kwon Jeong^3^, Jun Feranil^1,2^, Shaun C. Moore^1^, Xiaoxu Zheng^1^, Michael Bishop^1^, Jerald P. Gomes^2^, Jacob Polzin^1^, Noah Smeriglio^4^, Pedro A.S. Vaz de Castro^1^, [Ines Armando](https://pubmed.ncbi.nlm.nih.gov/?sort=date&size=200&term=Armando+I&cauthor_id=29080975)^1,2^,

[Robin A. Felder](https://pubmed.ncbi.nlm.nih.gov/?sort=date&size=200&term=Felder+RA&cauthor_id=19380616)^5^, Ling Hao^4^, Pedro A. Jose^1,2,3^

1. Department of Medicine, The George Washington University School of Medicine & Health Sciences, Washington, DC 20052.
2. Department of Medicine, University of Maryland School of Medicine, Baltimore, MD 21201
3. Department of Pharmacology and Physiology, The George Washington University School of Medicine & Health Sciences, Washington, DC 20052.
4. Department of Chemistry, Columbian College of Arts & Sciences, The George Washington University, Washington, DC 20052.
5. Department of Pathology, University of Virginia Health Sciences Center, Charlottesville, VA 22908.5

# Contributed equally

† Deceased.

***Address correspondence to:**

Hewang Lee, PhD

Department of Medicine

Division of Renal Diseases & Hypertension

The George Washington University

2300 Eye Street, NW

Washington, DC 20052

Tel: 202-994-2615

Email: lih@gwu.edu

**Running title:** Renal autocrine NPFF regulates blood pressure

**
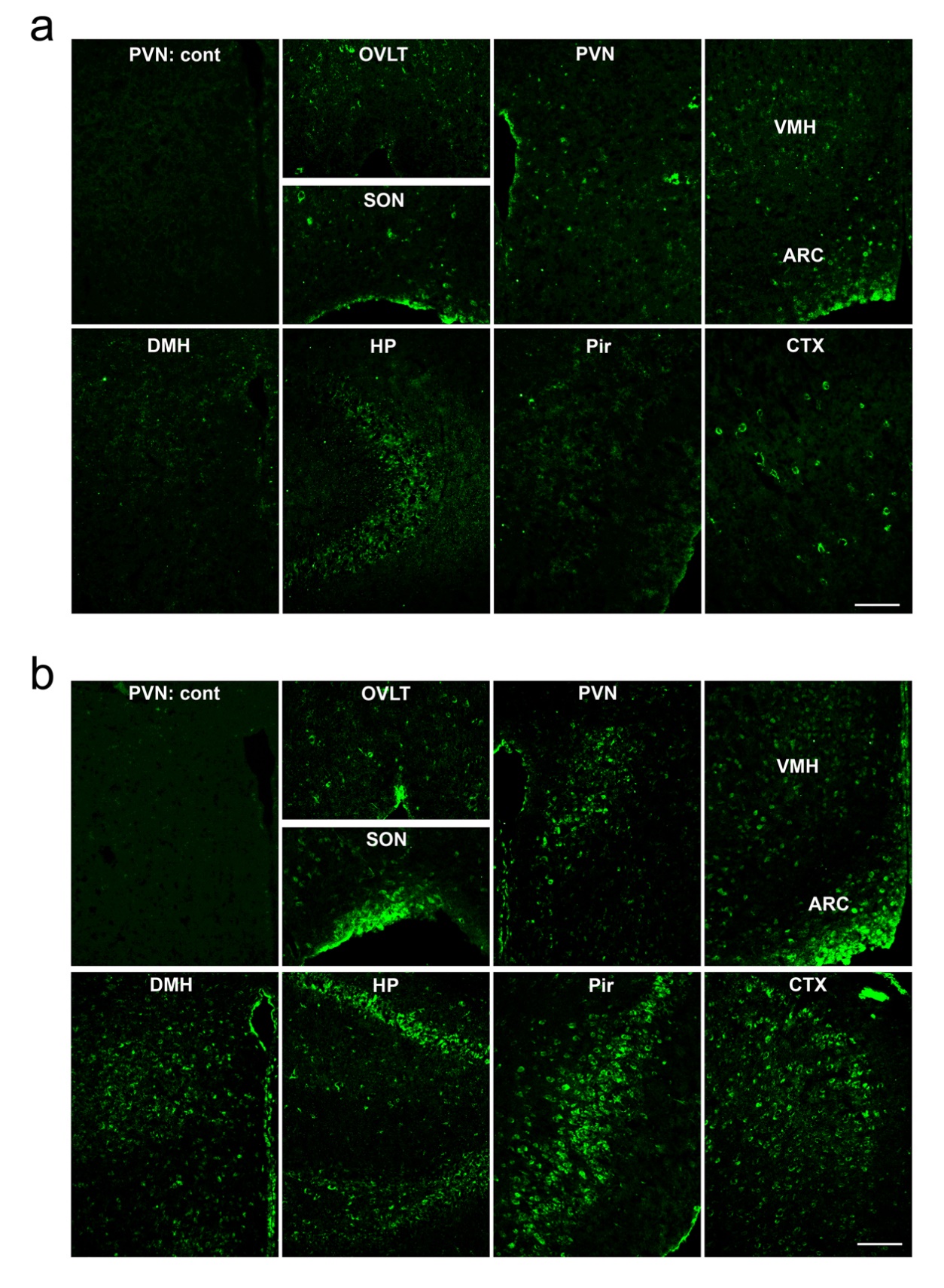
**

**Figure S1**: Immunostaining of NPFF-R1 (**a**) and NPFF-R2 (**b**) of mouse brain sections. Coronal sections of mouse brain were stained with anti-NPFF-R1 and anti-NPFF-R2 antibodies. NPFF-R1 immunoreactivity was detected in multiple hypothalamic nuclei including the organum vasculosum of the lamina terminalis (OVLT), supraoptic nucleus (SON), paraventricular nucleus (PVN), ventromedial hypothalamus (VMH) and arcuate nucleus (ARC), as well as in non-hypothalamic regions such as the hippocampus (HP) and cortex (CTX). However, NPFF-R1 immuno-signals were absent in the dorsomedial hypothalamus (DMH) and piriform cortex (Pir). By contrast, NPFF-R2 immunoreactivity was detected widely and strongly distributed throughout the entire mouse forebrain regions as shown in (**b**). Negative control, PVN: cont, showed no immunoreactivity for both antibodies. Bar scale, 100 µm.


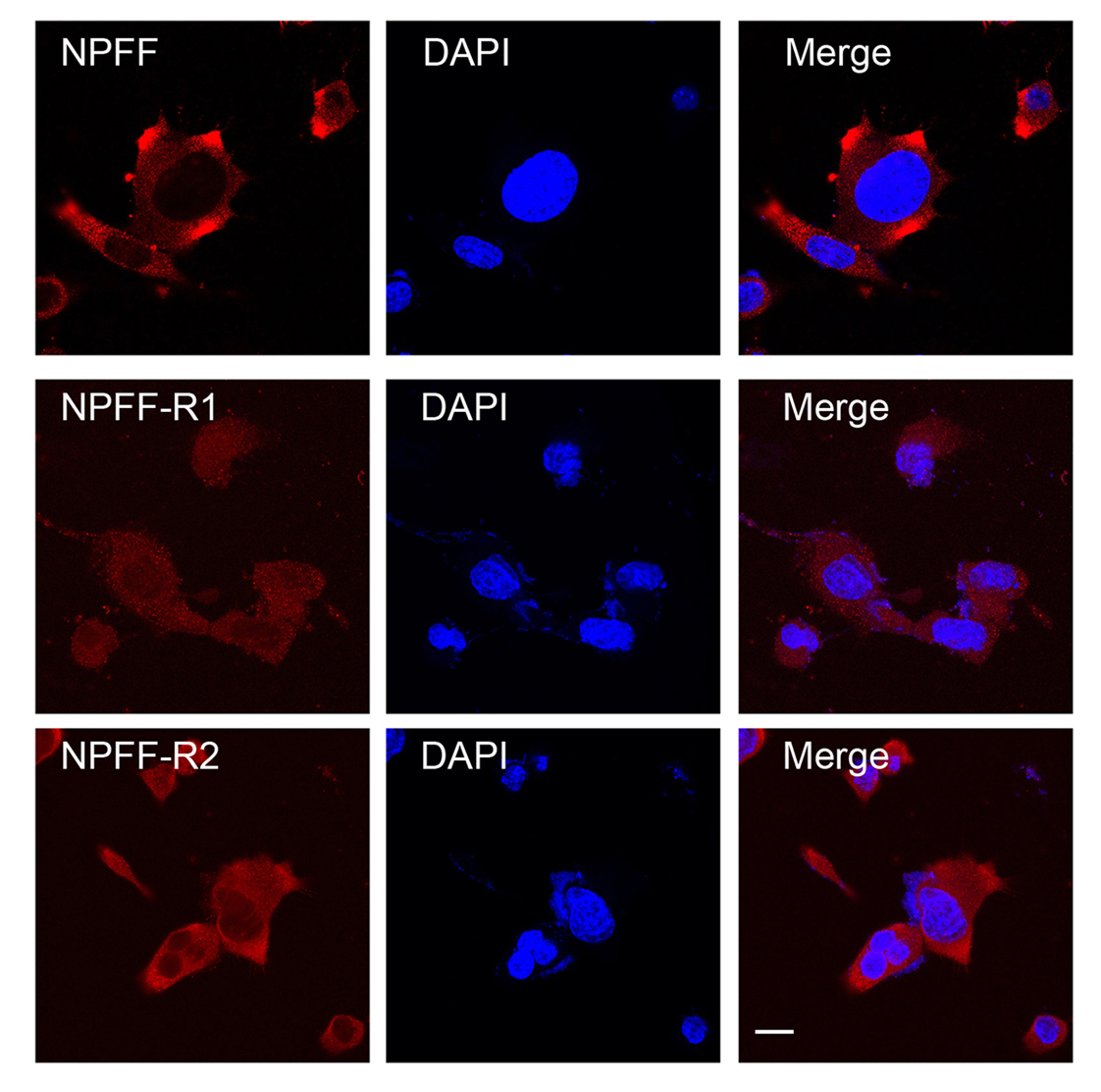


**Figure S2.** Protein expression of NPFF and its receptors in human renal proximal tubule cells (hRPTCs). hRPTCs were prepared and stained as described in the Materials and Methods section. The hRPTCs were incubated with anti-FMRF (for NPFF), anti-NPFF-R1, and anti-NPFF-R2 antibodies, as well as DAPI antibody to stain for DNA (nucleus), as shown. Bar scale, 10 μm.

**
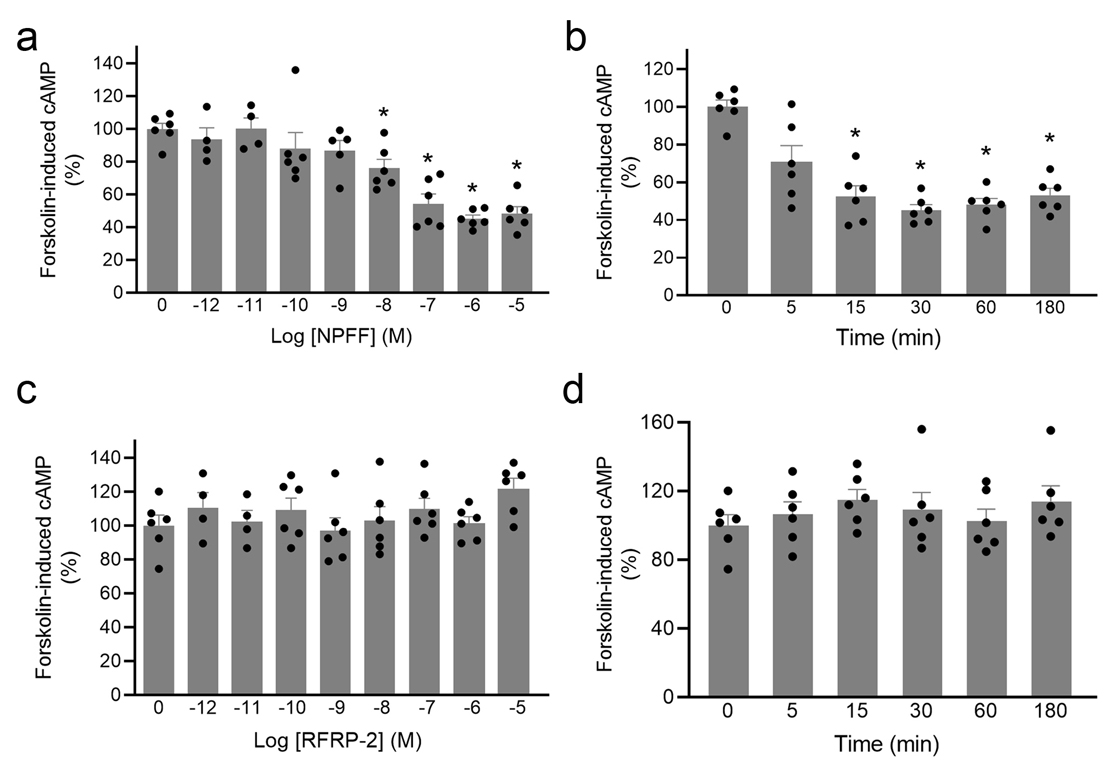
**

**Figure S3.** Inhibition of intracellular cAMP accumulation by NPFF in lysates of mouse (m) RPTCs. The cAMP concentration in lysates was determined by ELISA. (**a**) mRPTCs were exposed to the indicated concentrations of NPFF for 15 min, followed by 10 µM forskolin for 30 min. (**b**) mRPTCs were exposed to 10^-7^ M NPFF at the indicated time points, followed by10 µM forskolin for 30 min. (**c**) mRPTCs were exposed to the indicated concentrations of RFRP-2 for 15 min. (**d**) mRPTCs were exposed to RFRP-2 (10^-7^ M) at the indicated time points, followed by 10 µM forskolin for 30 min. n= 6/group, * *p* < 0.05 vs 0 M or 0 hr, one-way ANOVA, Newman-Keuls test.

**
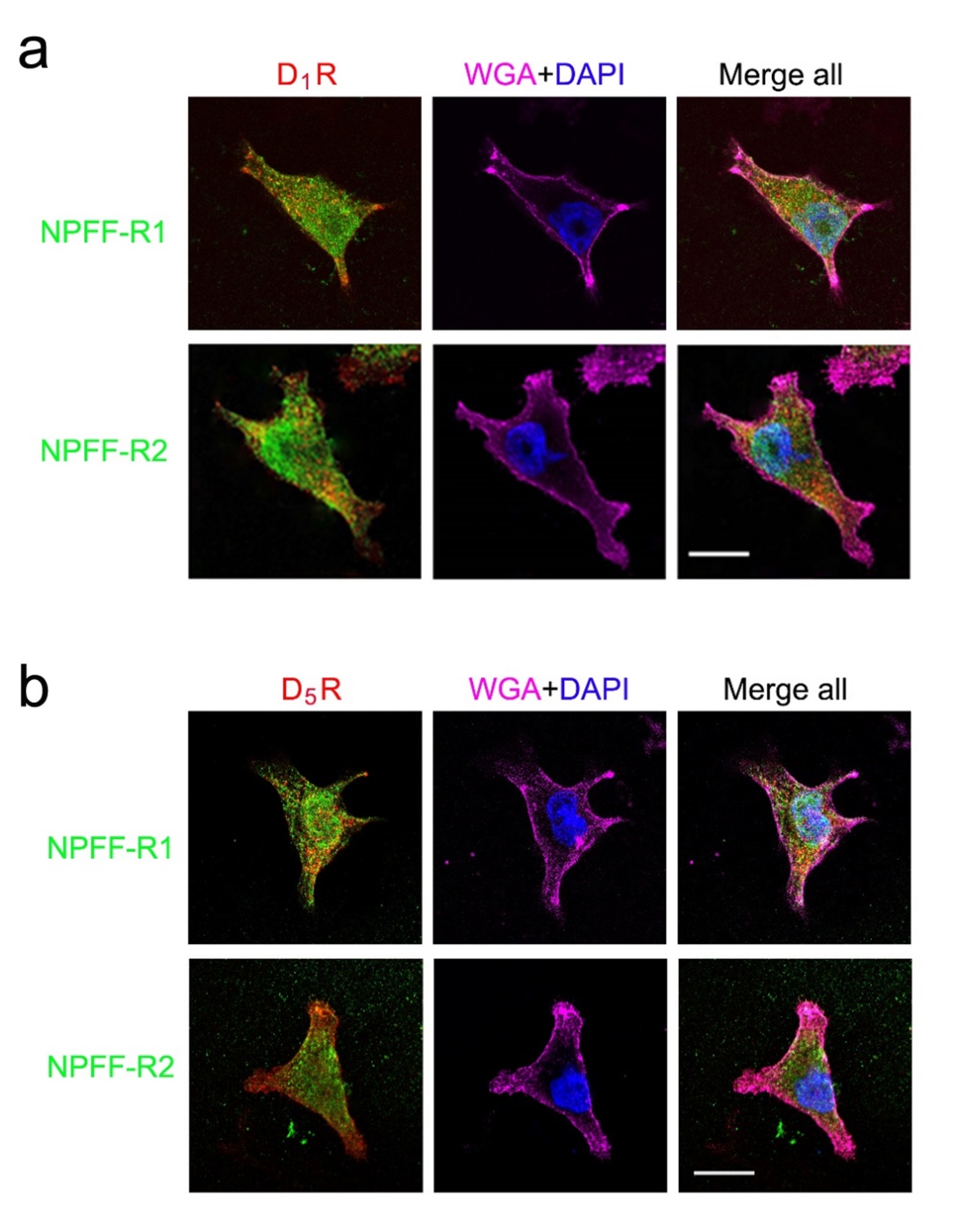
**

**Figure S4.** Colocalization of NPFF receptors with D1-like receptors (D_1_R and D_5_R) in hRPTCs. (**a**) Co-localization of NPFF-R1 and NPFF-R2 with D_1_R in hRPTCs. (**b**) Co-localization of NPFF-R1 and NPFF-R2 with D_5_R in hRPTCs. NPFF-R1 and NPFF-R2, green; D_1_R and D_5_R, red; wheat germ agglutinin (plasma membrane marker), magenta; DAPI (4',6-diamidino-2-phenylindole), blue. The colocalization of NPFF receptors with D_1_R or D_5_R is shown in yellow. Bar scale, 10 µm.

**
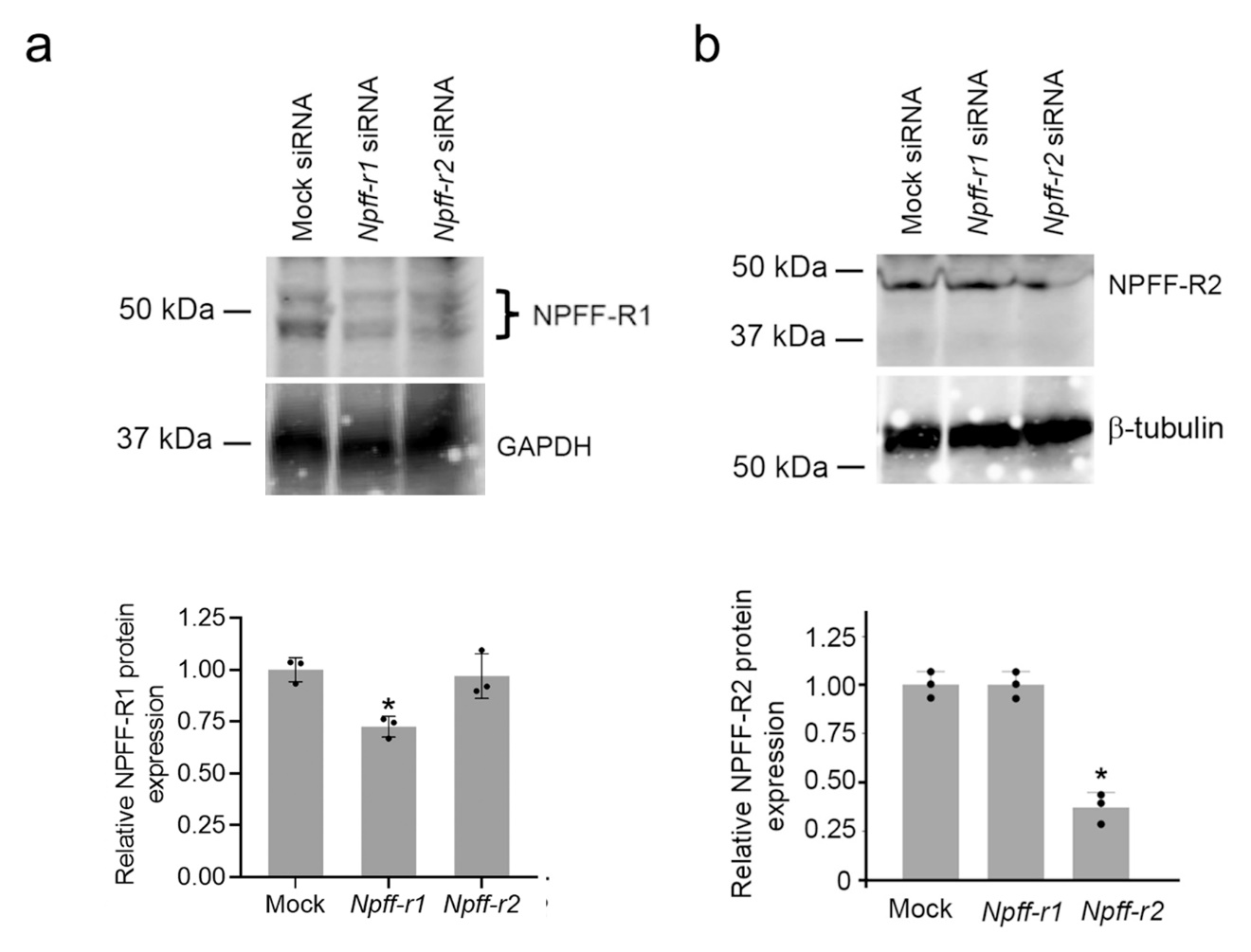
Figure S5**: Reduction of the protein expressions of NPFF-R1 and NPFF-R2 in renal cortices treated with the chronic renal subcapsular infusion of *Npff-r1* or *Npff-r2*-specific siRNA, respectively. The renal protein expressions of NPFF-R1 (**a**) and NPFF-R2 (**b**) were determined by immunoblotting in C57BL/6 mice fed a normal salt diet. Representative immunoblots of the protein expressions of NPFF-R1 (**a**) and NPFF-R2 (**b**) are shown above the bar graphs. The immunoblots of GAPDH and β-tubulin served as loading controls. n=3/group, * *p* < 0.05 vs Mock, one-way ANOVA, Newman-Keuls test.

**
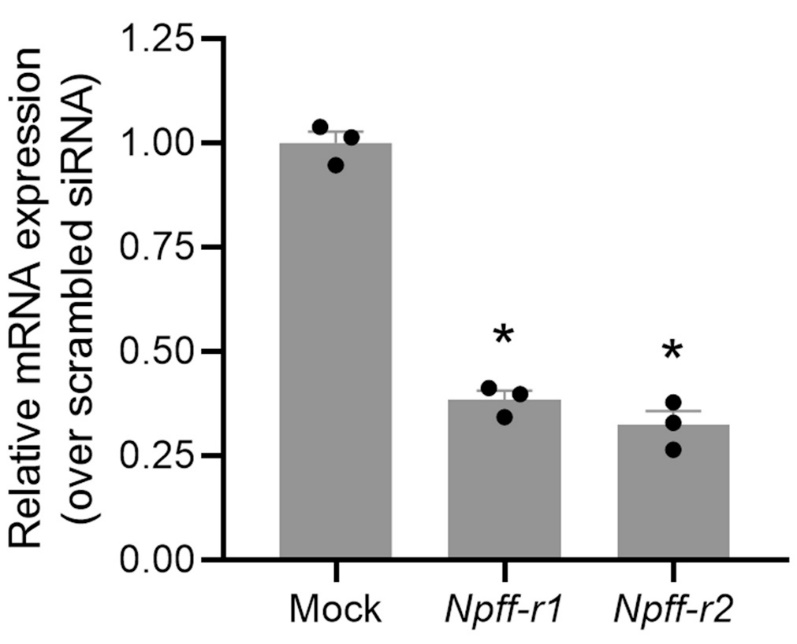
**

**Figure** **S6.** Reduction of the mRNA expressions of *Npff-r1 and Npff-r2* in renal cortices treated with the chronic renal subcapsular infusion of *Npff-r1 or Npff-r2*-specific siRNA. The renal mRNA expressions of *Npff-r1* and *Npff-r2* were quantified by qRT-PCR in C57BL/6 mice fed a normal salt diet. n=3/group, * *p* < 0.05 vs scrambled (Mock) siRNA, one-way ANOVA, Newman-Keuls test.

**Supplementary Table S1.** Primary antibodies used in this study

| Antibody | Source | Cat. No. | Immunogen | Species | Dilution |
| --- | --- | --- | --- | --- | --- |
| anti-D_1_R | Dr. Jose’s lab | N.A. | Human, aa351-446 (NP_000785.1); 88.7% identity, 97.9% similarity with mouse D_1_R | Rabbit, polyclonal | 1:200 |
| anti-D_5_R | Dr. Jose’s lab | N.A. | Human, aa272-285 (NP_000789.1); 71.4% identity, 78.6% similarity with mouse D_5_R | Chicken,  polyclonal | 1:100 |
| anti-NPFF | Millipore | ab15348 | Human, 100% identity with mouse NPFF | Rabbit, polyclonal | 1:200 |
| anti-NPFF-R1 | LifeSpan BioScience | LS-C61774 | Human, aa351-364 (NP_071429.1); 64.3% identity, 85.7% similarity with mouse NPFF-R1 | Goat, polyclonal | 1:100 |
| anti-NPFF-R1* | LifeSpan BioScience | LS-A1905 | Human, aa411-430 (NP_071429.1); 80.0% identity, 95.0% similarity with mouse NPFF-R1 | Rabbit, polyclonal | 1:100 |
| anti-NPFF-R2 | LifeSpan BioScience | LS-C135553 | Human, aa 251-300 (NP_004876.2); 82.0% identity, 96.0% similarity with mouse NPFF-R1 | Rabbit, polyclonal | 1:100 |
| anti-NHE3 | Sigma-Aldrich | MAB3136 | Rabbit, aa 703-832(NP_001076166.1);73.8% identity, 85.4% similarity with human NHE3;69.7% identity, 85.6% similarity with mouse NHE3 | Mouse, monoclonal | 1:150 |
| anti-phospho-NHE3 | Santa Cruz Biotechnology | [SC-53961](https://www.sigmaaldrich.com/US/en/product/mm/mabn2415) | Rat, phosphoserine 605, identity to mouse and human serine605 | Mouse, monoclonal | 1:150 |
| Anti-Na^+^/K^+^-ATPase | Millipore | 05-369 | Rabbit, α-1 subunit isoform | Mouse, monoclonal | 1:200 |
| Anti-GAPDH | Sigma-Aldrich | G8795 | Rabbit, GAPDH | Mouse, monoclonal | 1:1000 |

* Note: for mouse brain immunohistochemistry

**Supplementary Table S2.** Primers for RT-PCR of hRPTCs

| **Gene** | **Accession ID** | **Primer names** | **Primer sequences** | **Product length** |
| --- | --- | --- | --- | --- |
| *NPFF* | NM_003717.1 | NPFF _fwrd  NPFF _revs | 5’- TGCCTGCTCTAGATTCCCCTAA -3’  5’- CACCTACCCTCCTACAGCCAC -3’ | 92 |
| *NPFF-R1* | NM_022146.3 | NPFFR1_fwrd  NPFFR1_revs | 5'- GTGCTCAAGAACCGGCACAT-3’  5'- GCCACCCAGTGATGAGGTTG-3’ | 127 |
| *NPFF-R2* | NM_004885.1 | NPFFR2_fwrd  NPFFR2_revs | 5'- TGTACCTGGGTGCCCCTTAG -3’  5'- CAGAATCCACCAAGCCCGTC -3’ | 293 |

**Supplementary Table S3.** Primers for qRT-PCR of mouse kidneys

| **Gene** | **Accession ID** | **Primer names** | **Primer sequences** | **Product length** |
| --- | --- | --- | --- | --- |
| *Actb* | [NM_007393.3](https://www.ncbi.nlm.nih.gov/entrez/viewer.fcgi?db=nucleotide&id=145966868) | β-actin-fwrd  β-actin-revs | 5’-TGGCTCCTAGCACCATGAAG -3’  5’-AACGCAGCTCAGTAACAGTC -3’ | 193 |
| *Npff-r1* | [NM_001177511.1](https://www.ncbi.nlm.nih.gov/entrez/viewer.fcgi?db=nucleotide&id=1690504750) | Npff-r1_fwrd  Npff-r1_revs | 5'- CAACCTTATCACCGGTTGGC-3’  5'- AGGGACACATGATGAGCAGC-3’ | 218 |
| *Npff-r2* | NM_133192.2 | Npff-r2_fwrd  Npff-r2_revs | 5'- TGGCGGTTCTTTCTCATCCTC -3’  5'- GGGGCTGGTGGAGATAGTAGT -3’ | 215 |
| Mock1 |  | Mock1-fwrd  Mock1-revs | 5'- ACCGGCCTAGCTATTCCGTA -3’  5'- GCATGAGGCGAAGATAGCCA -3’ |  |
| Mock2 |  | Mock2-fwrd  Mock2-revs | 5'- GTCTTCTAGCCGTCTTCTGCT -3’  5'- AGGTGTATGGCGTGAGAGGTG -3’ |  |

**Uncropped images**
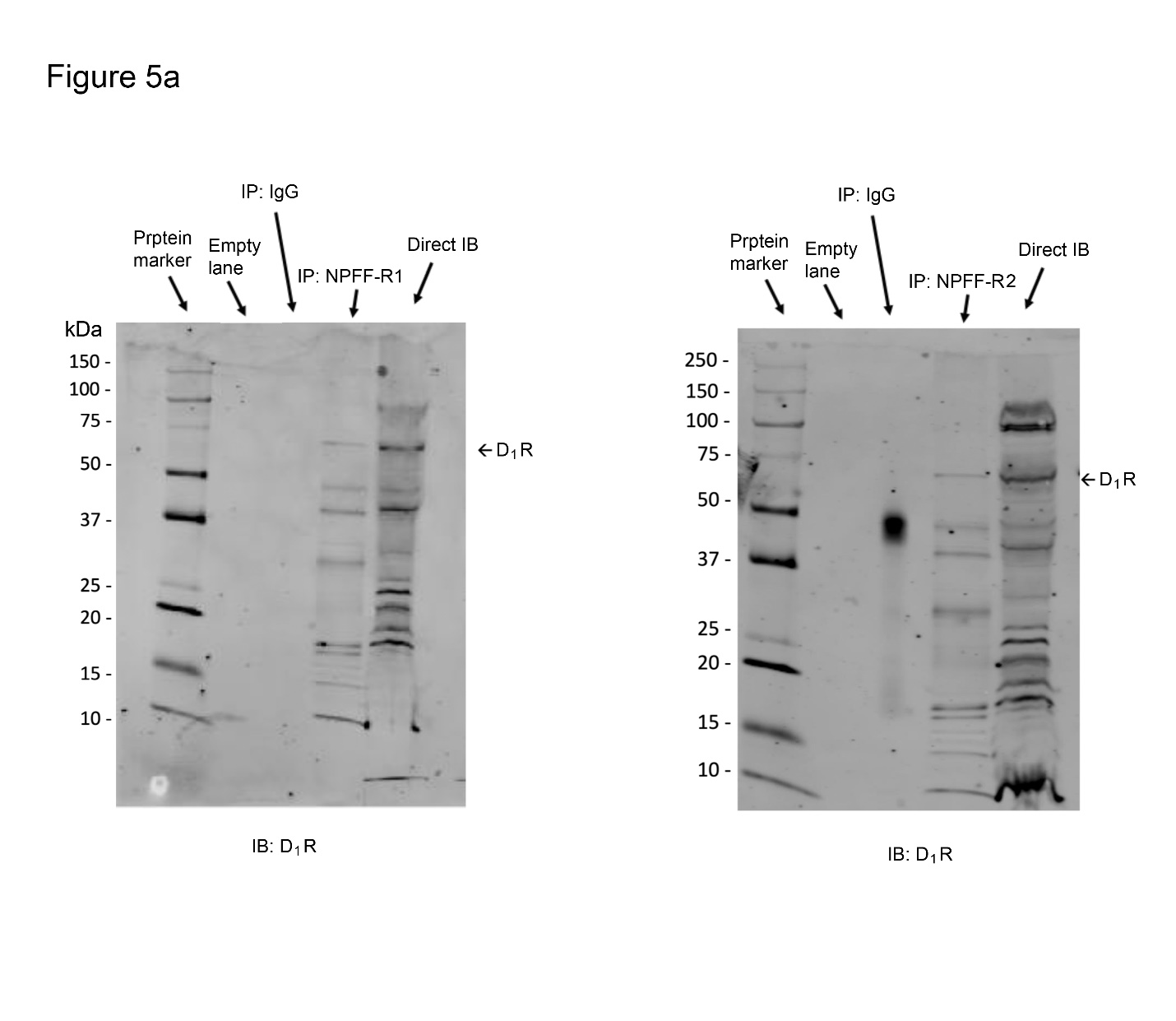
 **section**


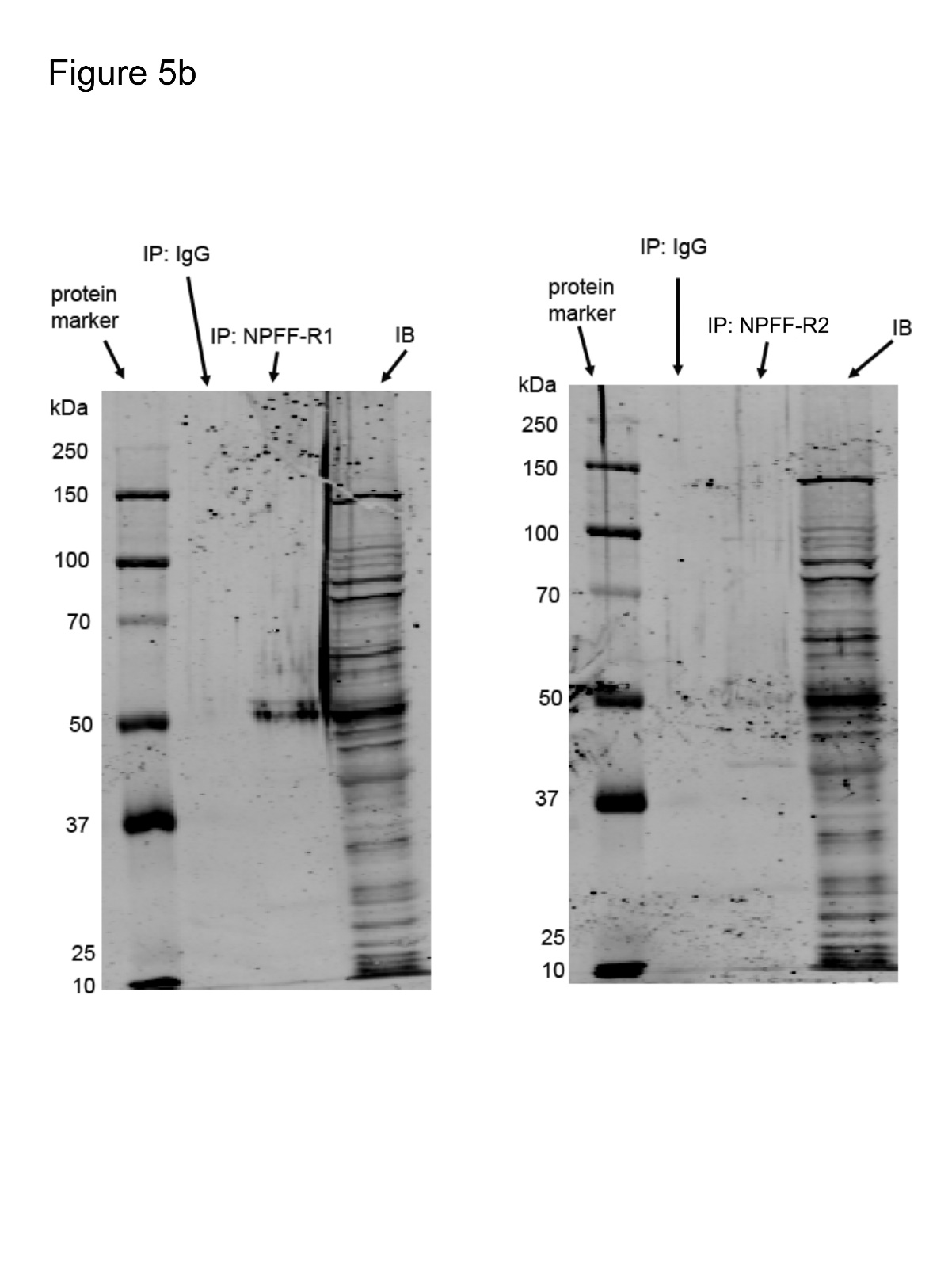


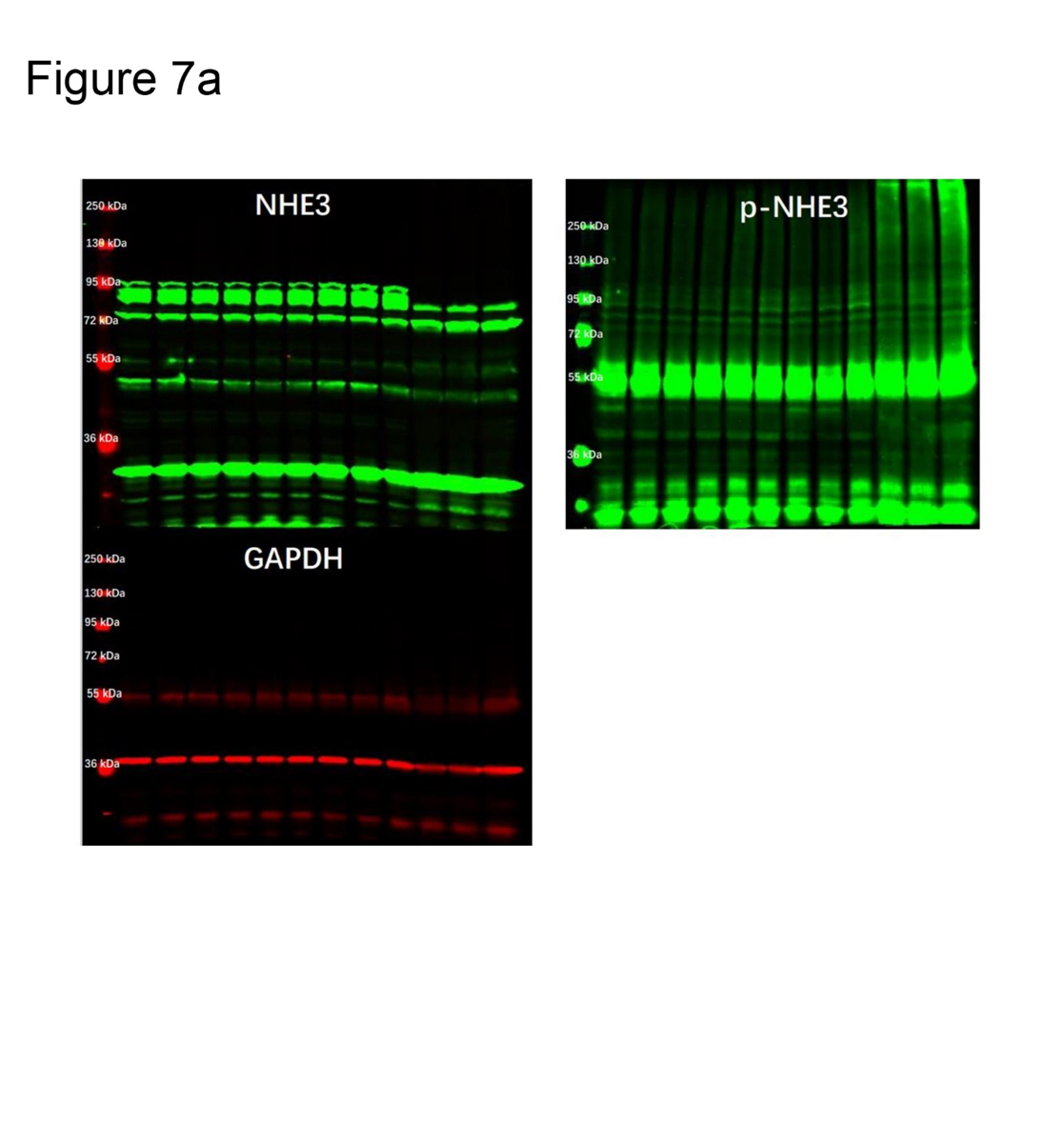


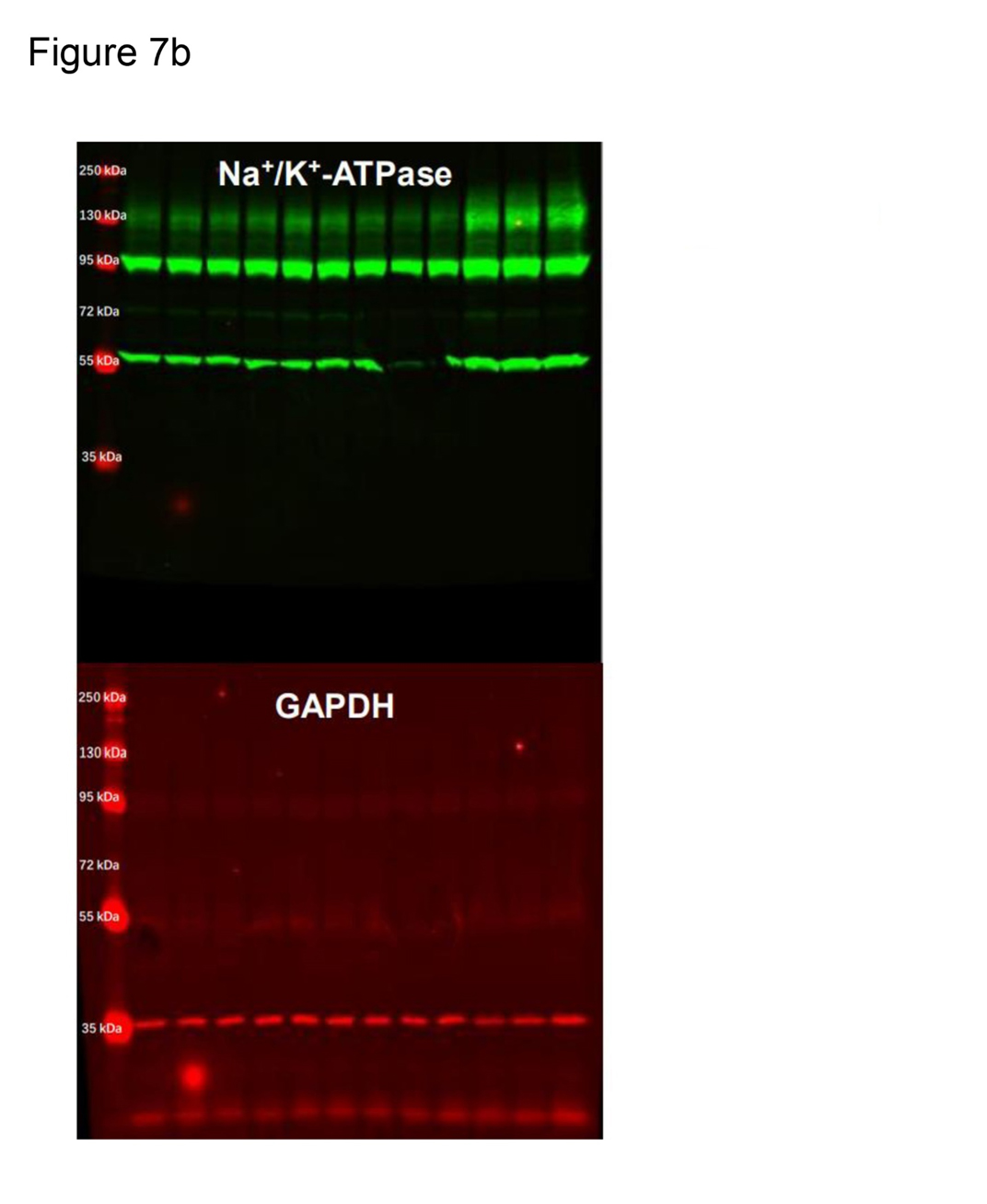


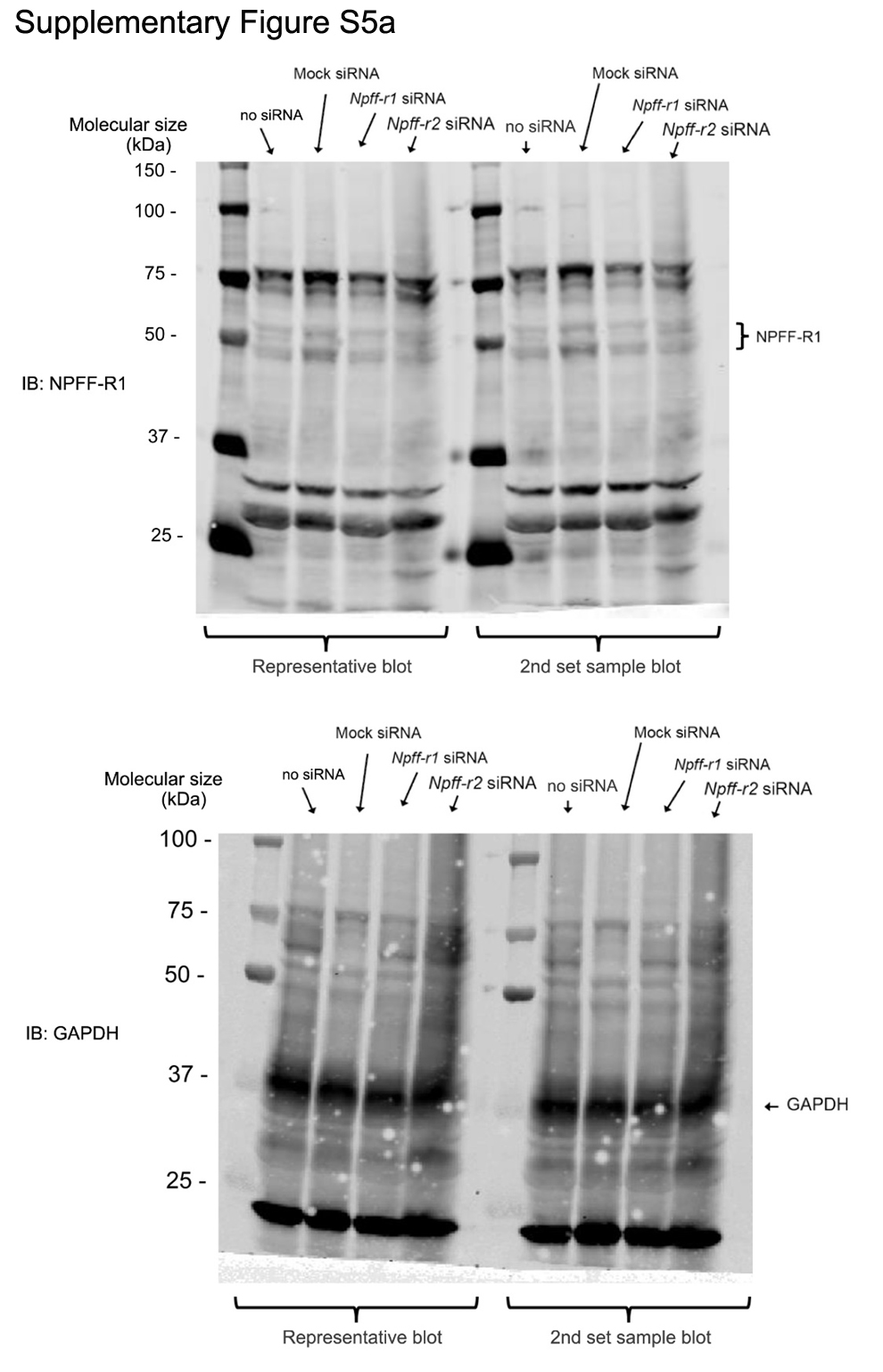


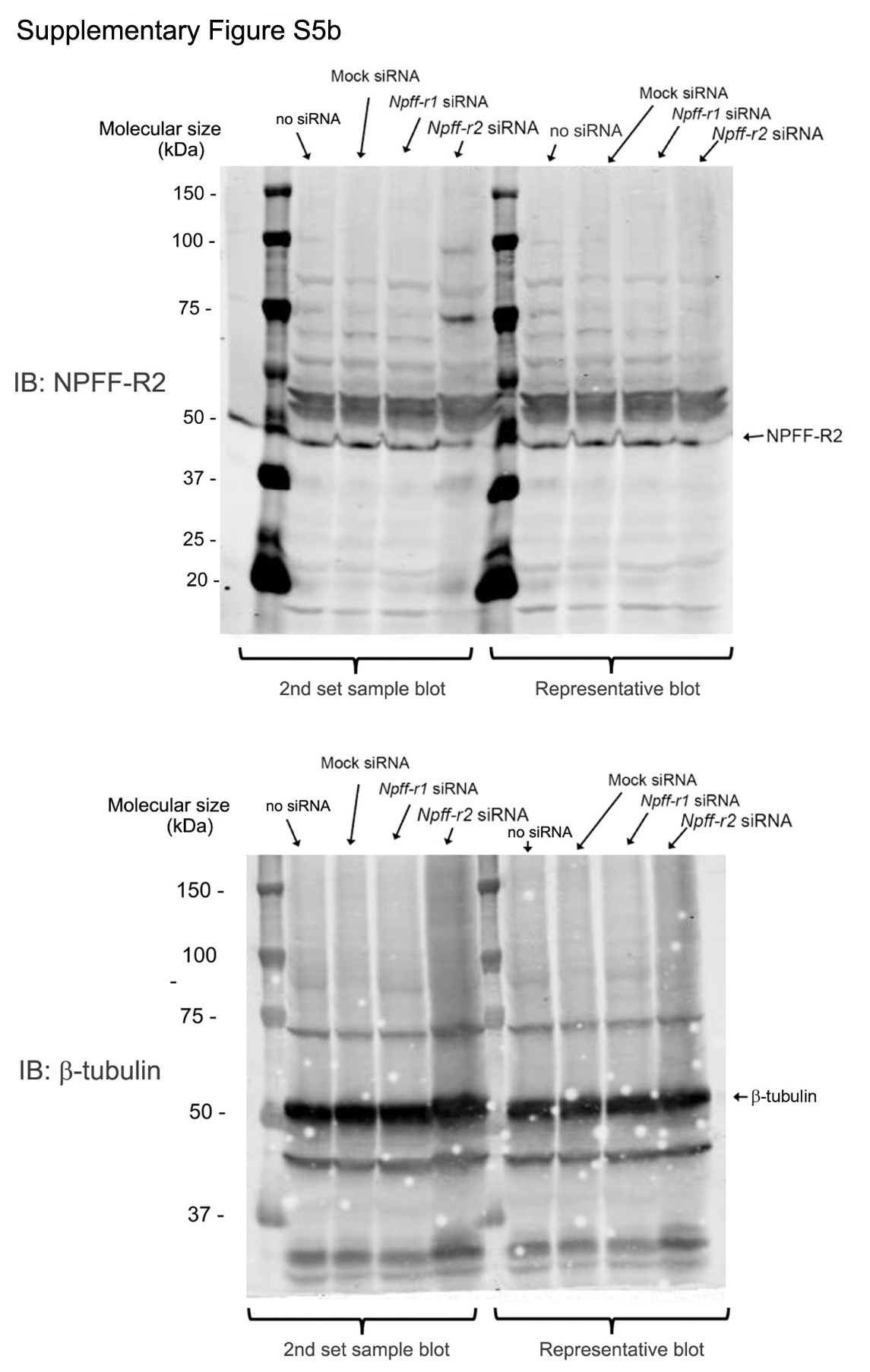

Supplement: Supplementary file 1 — Supplementary Information. [file 41598_2024_64484_MOESM1_ESM.docx]
